# Mettl3-mediated mRNA m^6^A methylation promotes dendritic cell activation

**DOI:** 10.1038/s41467-019-09903-6

**Published:** 2019-04-23

**Authors:** Huamin Wang, Xiang Hu, Mingyan Huang, Juan Liu, Yan Gu, Lijia Ma, Qi Zhou, Xuetao Cao

**Affiliations:** 1 Department of Immunology & Center for Immunotherapy, Institute of Basic Medical Sciences, Peking Union Medical College, Chinese Academy of Medical Sciences, 100005 Beijing, China; 2 National Key Laboratory of Medical Immunology & Institute of Immunology, Second Military Medical University, 200433 Shanghai, China; 3 College of Life Science, Nankai University, 300071 Tianjin, China; 4 Institute of Immunology, Zhejiang University School of Medicine, 310058 Hangzhou, China; 5 Westlake Institute for Advanced Study, 321116 Hangzhou, China; 6 State Key Laboratory of Reproductive Biology, Institute of Zoology, Chinese Academy of Sciences, 100101 Beijing, China

**Keywords:** Cell signalling, Antigen-presenting cells, Epigenetics in immune cells

## Abstract

N6-methyladenosine (m^6^A) modification plays important roles in various cellular responses by regulating mRNA biology. However, how m^6^A modification is involved in innate immunity via affecting the translation of immune transcripts remains to be further investigated. Here we report that RNA methyltransferase Mettl3-mediated mRNA m^6^A methylation promotes dendritic cell (DC) activation and function. Specific depletion of *Mettl3* in DC resulted in impaired phenotypic and functional maturation of DC, with decreased expression of co-stimulatory molecules CD40, CD80 and cytokine IL-12, and reduced ability to stimulate T cell responses both in vitro and in vivo. Mechanistically, Mettl3-mediated m^6^A of CD40, CD80 and TLR4 signaling adaptor Tirap transcripts enhanced their translation in DC for stimulating T cell activation, and strengthening TLR4/NF-κB signaling-induced cytokine production. Our findings identify a new role for Mettl3-mediated m^6^A modification in increasing translation of certain immune transcripts for physiological promotion of DC activation and DC-based T cell response.

## Abstract (web summary)

Here the authors examine how m^6^A modification is involved in innate immunity. They show that RNA methyltransferase Mettl3-mediated mRNA m^6^A methylation promotes dendritic cell (DC) activation and function, and in promoting DC-based T cells responses.

## Introduction

Post-transcriptional modifications of mRNAs, including N^6^-methyladenosine (m^6^A), 5-methylcytosine (m^5^C), and pseudouridine (ψ), are involved in the epigenetic regulation of multiple cellular processes, with broad roles in influencing mRNA stability, translation, and translocation^1–5^. As the most prevalent post-transcriptional modification on mammalian mRNA, the systemic and dynamic regulation of m^6^A is coordinated by multiple writer/eraser components. It is catalyzed mainly by a large RNA methyltransferase complex containing the methyltransferase-like (METTL) enzymes METTL3 and METTL14, Wilms tumor 1-associated protein (WTAP)^6,7^, among which METTL3 is in charge of catalyzing m^6^A formation, METTL14 is involved in binding target mRNA, and WTAP is responsible for the localization into nuclear speckles of the complex^7–9^; and removed by two demethylases: fat mass and obesity-associated protein (FTO) and α-ketoglutarate-dependent dioxygenase AlkB homolog 5 (ALKBH5)^10,11^. Recently, several new members are identified belonging to METTL3–METTL14–WTAP complex; RBM15/RBM15B is reported to recruit this complex to certain mRNA and lncRNA XIST to promote m^6^A formation^12^; KIAAI1429 and ZC3H3 are identified to regulate m^6^A methylation and play distinct functions in different cells^13,14^. Moreover, a group of m^6^A readers, such as YTH-domain family (YTHDF)1 and YTHDF2, could recognize m^6^A, and promote the translation and degradation of m^6^A-modified mRNAs, respectively^15,16^. Emerging evidence indicates that m^6^A modification regulates multiple biological pathways, such as stem cell differentiation, tumorigenesis, and viral replication by mediating RNA decay^17–20^. Recently, m^6^A is shown as an important mechanism of the host immune cell distinguishing self and non-self and also could be hijacked by a virus to evade immune response^21–23^; however, this role of m^6^A in innate immunity cannot be explained by a RNA degradation mechanism. Thus, it needs further investigation whether m^6^A has other physiological functions in mammalian cells, especially in immune cells, via RNA degradation-independent mechanism.

Dendritic cells (DC) are specialized antigen-presenting cells (APCs) linking innate and adaptive immune response^24–26^. DC is crucial for initiating adaptive immune responses for elimination of invading pathogens and also in inducing immune tolerance toward harmless components to maintain immune homeostasis^27,28^. The induction of immune activation or tolerance by DC strictly depends on distinct subsets of DCs at different maturation stages^29,30^. Generally, immature DC (imDC) induces immune tolerance, mature DC (maDC) stimulates and activates immune response, while regulatory DC (DCreg) downregulates immune responses via suppression of T-cell responses^31^. The dysregulation of DC activation at distinct stages is well known to be closely linked to multiple inflammatory, autoimmune, and other diseases^29,32^. Although transcriptional networks governing DC development and function have been intensively investigated^33–36^, the epigenetic mechanisms, especially the role of mRNA m^6^A modification in this process, remain to be fully understood. Therefore, identifying the role of m^6^A methylation in controlling DC activation is critical for better understanding of immune response and will also have important clinical implications.

It is well accepted that membrane co-stimulatory molecules, including CD40, CD80, and CD86, which could be upregulated by LPS stimulation, directly modulate the function and the stage of DC in their antigen presentation and T-cell activation. However, as multiple cells could respond to LPS stimulation, it is still unclear why only professional APCs, such as maDC, highly and continually express these co-stimulatory molecules after activation in response to innate stimuli. Molecular profiling of the imDC, maDC, and DCreg, the three kinds of DC at sequential but distinct maturation and differentiation stages, is a well-established cellular model for dynamically investigating the mechanisms of immune activation and regulation by DC at different subsets and stages^37^. In this study, we utilized DC maturation and a differentiation model, including bone marrow-derived imDC, LPS-stimulated BMDC (maDC), and DCreg, to investigate the expression patterns and biological roles of m^6^A modification in the maturation and function of DC. We demonstrate that mettl3 catalyzes m^6^A of CD40, CD80, and Tirap during DC maturation, and increases their translation efficiency to promote DC activation and function in promoting T-cell activation. Our study shed new light on the epigenetic regulation of innate immunity via m^6^A-mediated methylation of the related immune transcripts. In addition, our study provided a new mechanism why APC, once matured and activated, preferentially expresses a higher level of co-stimulatory molecules for efficiently initiating immune response.

## Results

### m^6^A modification level is increased during DC maturation

**Fig. 1:**
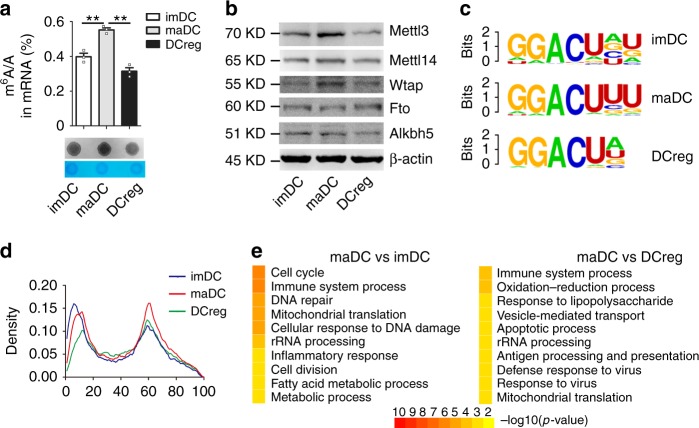
m^6^A modification level increases in DC maturation. **a** m^6^A level of imDC, maDC, and DCreg mRNA detected by HPLC–MS/MS (up) and dot-blot assays (down). **b** Indicated proteins level in imDC, maDC, and DCreg. **c** Sequence motifs identified within m^6^A peaks of imDC, maDC, and DCreg of two biological replicates with the lowest *p*-value. **d** Distribution of m^6^A peaks across the length of mRNA. Regions of 5’UTR, CDS, and 3′UTR are binned into 10, 50, and 40 segments according to the relative length, respectively, and the percentage of m^6^A peaks fall within each bin. **e** GO enrichment analysis in the biological process category of transcripts bearing m^6^A modification only in maDC compared with that in imDC (left) or that in DCreg (right). Data are shown as mean±SEM (up panel of a) of three determinants, and from one representative of three independent experiments (down panel of a, b). *P < 0.05 and **P < 0.01 (Student’s *t-*test, two-tailed)

We first investigated the abundance of m^6^A and m^6^A writers/erasers in the three sequential but distinct DC subsets: bone marrow-derived imDC, LPS-stimulated BMDC (maDC), and DCreg. HPLC–MS/MS and dot-blot experiments revealed that the m^6^A modification level was significantly increased in maDC compared with that in imDC and DCreg (Fig. 1a). In line with this dynamic change of m^6^A modification level, m^6^A methyltransferases Mettl3, Mettl14, and Wtap were also increased in maDC (Fig. 1b). We then profiled the transcriptome-wide mRNA m^6^A modification in two replicates of imDC, maDC, and DCreg using m^6^A immunoprecipitation together with high-throughput sequencing (meRIP-seq). The correlations between two biological replicates were mostly over 0.85, which shows a strong correlation. High-confidence m^6^A peaks were enriched in transcripts of 6004, 7990, and 6624 genes in imDC, maDC, and DCreg, respectively. Further motif enrichment analysis revealed that m^6^A peaks identified above shared a common sequence element GGACU (Fig. 1c) and the CDS segment harbored the largest fraction of peaks (Supplementary Fig. 1a). A metagene profile revealed m^6^A peaks around the transcription start site and stop codon site in DC (Fig. 1d), consistent with previous studies^38^. In line with the increase of the m^6^A level in maDC, m^6^A peaks were enriched in more transcripts in maDC, which were clustered in the immune system and inflammation response as analyzed by GO enrichment (Fig. 1e). Specifically, multiple newly appeared m^6^A peaks in maDC were enriched in transcripts of NOD-like receptor (NLR) signaling pathway and TNF signaling pathway (Supplementary Fig. 1b) when compared with imDC, while enriched in transcripts of the TNF signaling pathway and NF-κB signaling pathway when compared with DCreg (Supplementary Fig. 1b). NF-κB is the major downstream regulator of NLR, TNF signaling pathway and plays a pivotal role in the induction of co-stimulatory molecules and proinflammatory cytokines during DC maturation; these data hint a potential role of m^6^A in immune response of maDC, especially in the NF-κB activation.

### Mettl3 promotes DC maturation in a m^6^A catalytic activity-dependent manner

m^6^A modification is catalyzed by a RNA methyltransferase complex, while Mettl3 functions as the predominant catalytic subunit^8,9^. Previous studies showed that knockdown of *Mettl3* resulted in apoptosis of human HeLa cells and HepG2 cells, and disruption of *Mettl3* homologs led to a lethal phenotype in mice^7,17,38^. To further explore the role of m^6^A in DC maturation and function in vivo, we generated *Mettl3* conditional knockout mice with specific deletion of *Mettl3* exon2~4 in DC by Cre recombinase expressed from the DC-specific *CD11c* promoter (*Mettl3*^fl/fl^CD11c-Cre mice). The mRNA and protein levels of Mettl3 were both abolished in *Mettl3*^fl/fl^ CD11c-Cre (*Mettl*3KO) DC (Supplementary Fig. 2a, b). Moreover, the m^6^A level was significantly decreased in *Mettl3*KO DC as compared with *Mettl3*^fl/fl^ (*Mettl3*WT) DC (Supplementary Fig. 2c). The frequency of CD11c-positive cells in the splenocytes and bone marrow-derived DC (BMDC) from *Mettl3*WT mice and *Mettl3*KO mice was similar (Supplementary Fig. 2d). In addition, no apoptosis was observed in BMDC, either from *Mettl3*WT or *Mettl3*KO mice (Supplementary Fig. 2e). These data indicate that deficiency of *Mettl3* did not affect DC generation from precursor cells or its apoptotic process.

**Fig. 2:**
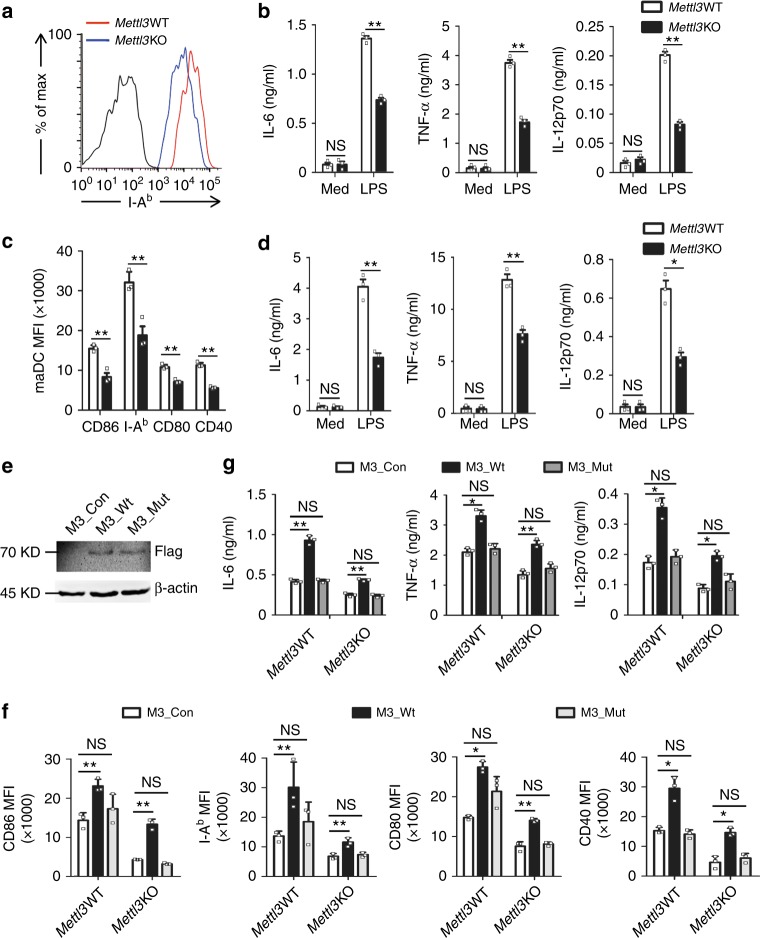
Mettl3 promotes DC maturation in a m^6^A catalytic activity-dependent manner. **a** Expression of I-A^b^ of LPS-stimulated *Mettl3*WT and *Mettl3*KO splenic DC. **b** Cytokines in supernatants of splenic *Mettl3*WT and *Mettl3*KO DC treated with medium alone (Med) or LPS (100 ng/ml) for 24 h. **c** Expression of phenotypic markers of *Mettl3*WT and *Mettl3*KO maDC shown as median fluorescence intensity (MFI). **d** Cytokines in supernatants of *Mettl3*WT and *Mettl3*KO BMDC treated with medium alone (Med) or LPS (100 ng/ml) for 24 h. **e** Expression of Flag or β-actin in the whole-cell lysates of *Mettl3*KO BMDC transfected with lentiviruses overexpressing control (M3_Con) or Flag-tagged wild-type Mettl3 (M3_Wt) or catalytic mutation of Mettl3 (M3_Mut). **f** Expression of surface markers of *Mettl3*WT and *Mettl3*KO maDC transfected with lentiviral vectors overexpressing control (M3_Con) or wild-type Mettl3 (M3_Wt) or catalytic mutation of Mettl3 (M3_Mut). **g** ELISA of cytokines in supernatants of *Mettl3*WT and *Mettl*3KO DC treated as in **f**. Data are from one representative of three independent experiments (**a**, **e**) and shown as mean±SD (**c**, **f**, and **g**) or SEM (**b**, **d**) of three determinants. *P < 0.05, **P < 0.01, NS, not significant (Student’s *t*-test, two-tailed)

Next, we sought to examine the effect of *Mettl3* deficiency on DC activation and function. As shown in Fig. 2a, b and Supplementary Fig. 3a, *Mettl3*KO splenic DC had decreased expression of MHC class II (I-A^b^) and co-stimulatory molecules CD86, CD80, and CD40, and impaired production of proinflammatory cytokines IL-6, TNF-α, and IL-12p70 in response to LPS stimulation. The decreased expression of CD86, I-A^b^, CD80, and CD40 was also observed in *Mettl3*KO splenic DC after *Listeria monocytogens* infection (Supplementary Fig. 3b). *Mettl3*KO BMDC also expressed lower levels of CD86, I-A^b^, CD80, and CD40, and produced less IL-6, TNF-α, and IL-12p70 in response to LPS stimulation (Fig. 2c, d). To explore whether the impaired phenotype and cytokine production of *Mettl3*KO DC was caused by disruption of m^6^A modification, we performed rescue experiments in *Mettl3*KO DC by transfection with lentiviruses encoding wild-type *Mettl3* (M3_Wt) or catalytic mutant *Mettl3* (D394A and W397A, M3_Mut)^39^, respectively (Fig. 2e). While overexpression of M3_Wt could restore the expression of CD86, I-A^b^, CD80, and CD40 and the secretion of IL-6, TNF-α, and IL-12p70 in LPS-stimulated *Mettl3*KO DC, overexpression of a catalytic mutation M3_Mut had no such effects (Fig. 2f, g). These data indicate that Mettl3 promotes the maturation phenotype and proinflammatory cytokine secretion of DC, depending on its m^6^A catalytic activity.

### Mettl3 promotes DC function in T-cell activation in a m^6^A catalytic activity-dependent manner

**Fig. 3:**
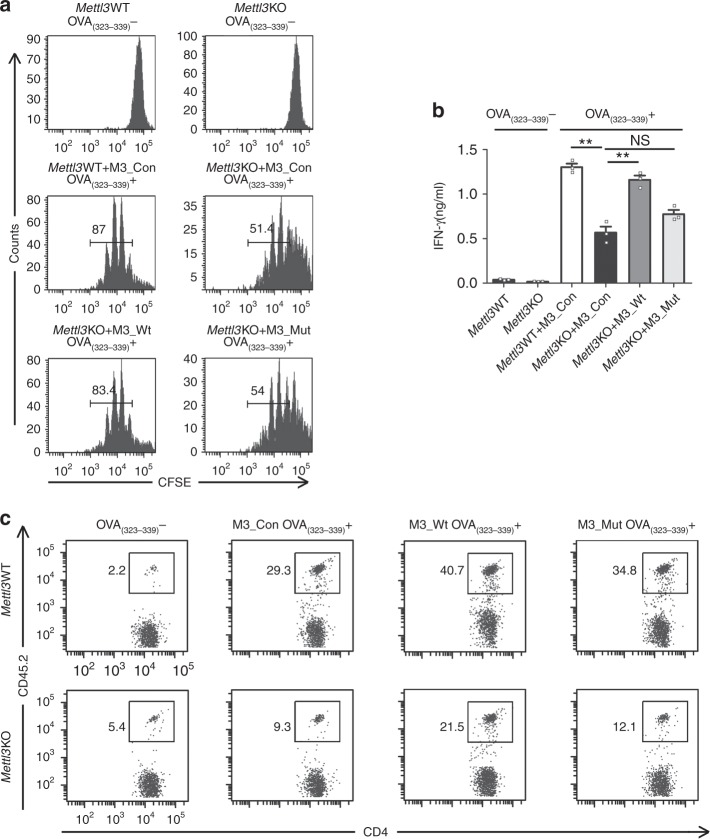
Mettl3 promotes DC function in priming T cells in a m^6^A catalytic activity-dependent manner. **a** Proliferation of CFSE-labeled OT-II CD4^+^ T cells incubated with *Mettl3*WT DC transfected with M3_Con or *Mettl3*KO DC transfected with M3_Con, M3_Wt, and M3_Mut and then pulsed with or without OVA_(323–339)._ **b** IFN-γ production of CFSE-labeled OT-II CD4^+^ T cells incubated with *Mettl3*WT DC transfected with M3_Con or *Mettl3*KO DC transfected with M3_Con, M3_Wt, and M3_Mut pulsed with or without OVA_(323–339)._ **c** In vivo proliferation of CD45.2^+^CD4^+^ OT-II T cells in recipient mice immunized with *Mettl3*WT or *Mettl3*KO DC, which were transfected with M3_Con, M3_Wt, and M3_Mut  and then pulsed with or without OVA_(323–339)._ Data are from one representative of three independent experiments (**a**, **c**) and shown as mean±SEM (**b**) of three determinants. **P < 0.01, NS, not significant (Student’s *t-*test, two-tailed)

We next analyzed the function of *Mettl3*KO DCs to promote T-cell proliferation in vitro and in vivo. As expected, *Mettl3*KO maDC had an impaired ability to initiate the proliferation and IFN-γ production of allogeneic CD4^+^ T cells, which could be restored by overexpression of M3_Wt but not M3_Mut in vitro (Fig. 3a, b). Furthermore, we transferred the recipient CD45.1^+^ wild-type mice with CD45.2^+^CD4^+^OT-II T cells, followed by immunization with unpulsed or OVA_(323–339)_-pulsed *Mettl3*WT or *Mettl3*KO DC 1 day later. The abundance of CD45.2^+^CD4^+^ T cells in the draining popliteal lymph nodes was analyzed by flow cytometry 4 days later. Consistent with the in vitro experiment, immunization with *Mettl3*KO DC resulted in significantly reduced proliferation of CD45.2^+^CD4^+^ T cells in the recipient mice, only one-third of that in recipient mice immunized with *Mettl3*WT DC (Fig. 3c). Overexpression of M3_Wt, but not M3_Mut, in OVA_(323–339)_-pulsed *Mettl3*WT and *Mettl3*KO DC both induced a larger amount of CD45.2^+^CD4^+^ T cells in the recipient mice (Fig. 3c). These data indicate that Mettl3 is required for DC function in promoting T- cell proliferation via its m^6^A catalytic activity, both in vitro and in vivo.

### Mettl3 strengthens innate response and NF-κB signaling during DC maturation

**Fig. 4:**
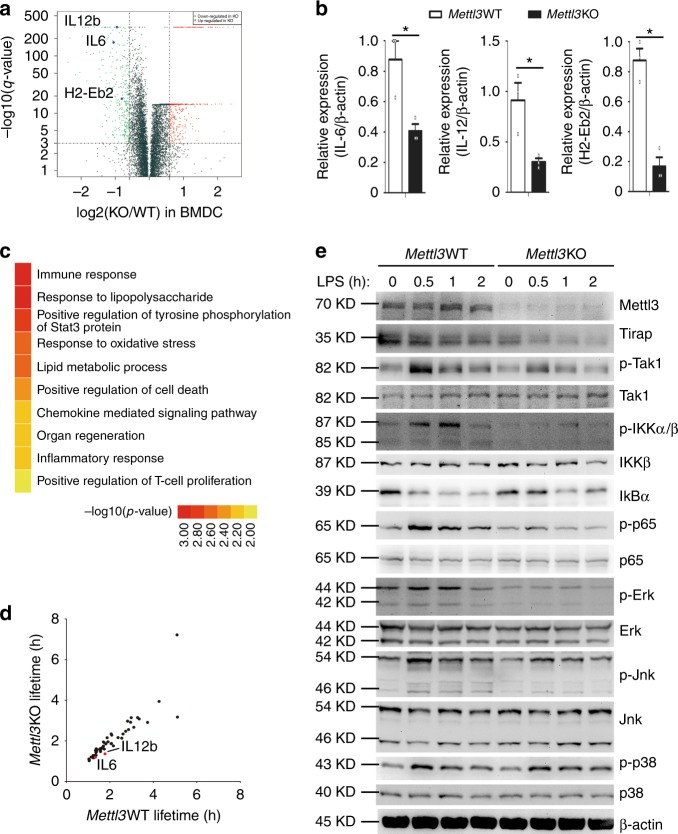
Mettl3 strengthens innate response and NF-κB signaling during DC maturation. **a**, **b** RNA-seq (**a**) and qPCR (**b**) shows that IL-6, IL-12, and H2-Eb2 are among the most significantly downregulated genes in LPS-stimulated *Mettl3*KO over *Mettl3*WT DCs. **c** GO enrichment analysis of transcripts with more than 1.5-fold expression in *Mettl3*WT DC than that in *Mettl3*KO DC in the biological process category. **d** Calculated lifetime of the downregulated genes as in **c**. **e** Expression of phosphorylated (p-) and total signaling proteins in whole-cell lysates of *Mettl3*WT and *Mettl3*KO DC stimulated with LPS for various times (above lanes). Data are from one representative of three independent experiments (**e**) and shown as mean ± SEM (**b**) of three determinants. *P < 0.05 (Student’s *t* test, two-tailed)

Next, we sought to investigate the molecular mechanism of Mettl3 in promoting DC maturation and activation via m^6^A modification. We performed RNA-sequencing (RNA-seq) analysis on maDC from *Mettl3*KO mice and littermate *Mettl3*WT control mice. Consistent with our observations, deficiency of *Mettl3* caused downregulation of the downstream effector molecules of the TLR4/NF-κB pathway, such as MHC class II molecule H2-Eb2, cytokines IL-6 and IL-12b mRNA level (Fig. 4a), which was also validated by qPCR (Fig. 4b). In both replicates of RNA-seq, the downregulated transcripts (Supplementary Table 1) in *Mettl3*KO DC compared with *Mettl3*WT DC were enriched in immune responses, especially the innate inflammatory response to LPS (Fig. 4c). Since multiple studies have revealed the association between m^6^A and mRNA decay, we first wondered whether the decreased IL-6, IL-12b, and H2-Eb2 mRNA level resulted from accelerated mRNA decay. We conducted a genome-wide measurement for mRNA stability change in maDC of *Mettl3*WT and *Mettl3*KO mice. However, there was no difference in the lifetime of all the downregulated genes in *Mettl3*KO DC, including IL-6, IL-12b, and H2-Eb2 (Fig. 4d), among which IL-6 and H2-Eb2 had m^6^A modification peaks (Supplementary Fig. 4a). RNA decay assays confirmed the degradation level of IL-6 and IL-12b, and H2-Eb2 mRNA was comparable between *Mettl3*WT DC and *Mettl3*KO DC (Supplementary Fig. 4b). These data suggested that the decreased IL-6 and IL-12 mRNA level in *Mettl3*KO DC may result from decreased transcription activity rather than accelerated mRNA degradation.

We thus suspected that *Mettl3*KO DC might have a defect in TLR4/NF-κB signaling pathway, which had caused the decreased IL-6 and IL-12 mRNA level. We measured the protein expression and phosphorylation level of NF-κB pathway signaling molecules. As expected, upon LPS stimulation, *Mettl3*KO DC had significantly decreased phosphorylation of the signaling molecules TAK1, IKKα, IKKβ, ERK, JNK, and the NF-κB subunit p65, than did *Mettl3*WT DC (Fig. 4e). Notably, Tirap, an important adaptor in the TLR4/NF-κB signaling pathway promoting TLR4 recruitment of Myd88, upstream of TAK1 in the signaling pathway, had a lower protein level in LPS-stimulated *Mettl3*KO DC (Fig. 4e), which indicated that Mettl3 might promote the NF-κB signaling through Tirap during DC maturation and activation.

### Mettl3 promotes the translation of Tirap, CD80, and CD40 mRNA in vivo

**Fig. 5:**
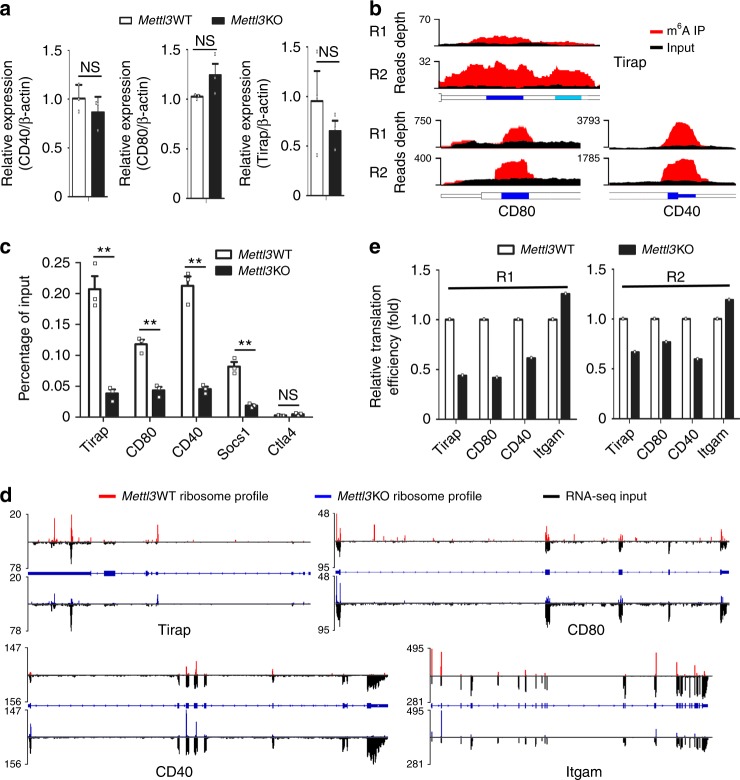
Mettl3 promotes the translation of Tirap, CD80, and CD40 mRNA in vivo. **a** Indicated mRNA expression in *Mettl3*WT or *Mettl3*KO maDC. The results are normalized by mouse β-actin and presented relative to those of *Mettl3*WT mature DC, set as 1. **b** m^6^A peaks marked in blue are enriched in the 3′UTRs of Tirap and CD40, CDS of CD80 genes from m^6^A RIP-seq data in maDC. R1 and R2 were representative of meRIP replicate1 and replicate2. **c** m^6^A RIP-qPCR shows that Tirap, CD80, and CD40 are m^6^A modified in *Mettl3*WT maDCs rather than those in *Mettl3*KO maDC. **d** Ribosome profiles of Tirap, CD80, and CD40 mRNAs. **e** Calculated translation efficiency of the indicated genes shows that Tirap, CD80, and CD40 are translationally decreased in *Mettl3*KO maDC. R1 and R2 were representative of ribosome profile replicate1 and replicate2. Data from one representative of two independent replicates (**e**) and shown as mean±SEM (**a**, **c**) of three determinants. **P < 0.01, NS, not significant (Student’s *t* test, two-tailed)

We next wondered whether Mettl3 regulates the expression of Tirap, CD80, and CD40 via m^6^A-dependent mechanisms. We found that Tirap mRNA level was similar in *Mettl3*KO DC compared with *Mettl3*WT DC (Fig. 5a), CD40 and CD80 had similar mRNA level but decreased protein level in *Mettl3*KO DC (Fig. 5a). All these three molecules (Tirap, CD40, and CD80) were m^6^A modified in maDC (Fig. 5b), and the m^6^A modification level was greatly reduced in *Mettl3*KO maDC, using Socs1 m^6^A as the positive control, which has been identified in DC and T cells previously^40^ (Fig. 5c and Supplementary Fig. 5). As m^6^A modification has been reported to affect mRNA translation in some studies^16,41^, we suspected whether Mettl3 promotes the protein expression of Tirap, CD80, and CD40 at translation levels. So we performed two replicates of genome-wide ribosome profiling to detect mRNA translation efficiency. We found that Tirap, CD80, and CD40 all had a lower translation efficiency in *Mettl3*KO maDC compared with that in *Mettl3*WT maDC (Fig. 5d, e), which was verified by qPCR of ribosome-associated RNA separated from 80 S monosome fraction of *Mettl3*WT and *Mettl3*KO maDC lysates (Supplementary Fig. 6). These results indicated that the mRNA of Tirap, CD80, and CD40 had a decreased translation efficiency in *Mettl3*KO maDCs in vivo.

### Mettl3 promotes the translation of Tirap, CD80, and CD40 mRNA in vitro

**Fig. 6:**
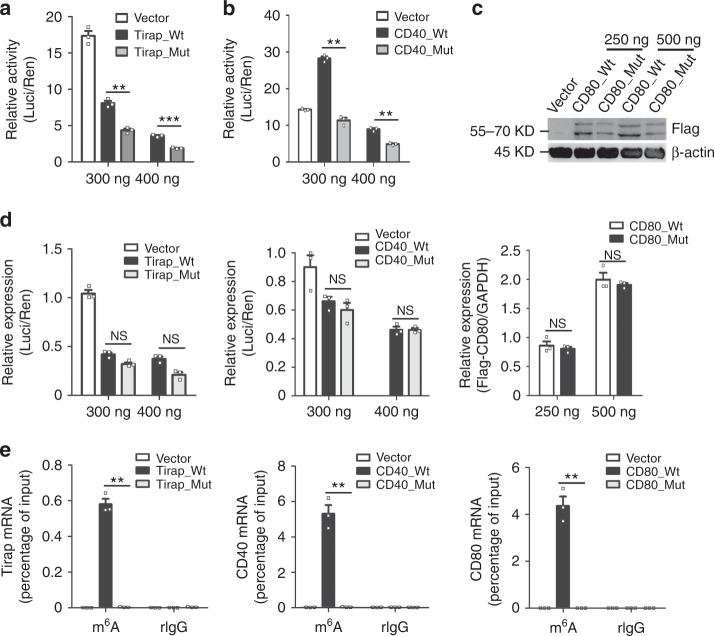
Mettl3 promotes the translation of Tirap, CD80, and CD40 mRNA in vitro. **a**, **b** Relative luciferase activity of pMIR-REPORT with empty pMIR (Vector), wild-type, or the m^6^A site mutation of Tirap-3′UTR (Tirap_Wt and Tirap_Mut) (**a**) or of CD40-3′UTR (CD40_Wt and CD40_Mut) (**b**) transfected into HEK293T cells. Firefly luciferase activity (Luci) was measured and normalized to Renilla luciferase activity (Ren). **c** Flag expression in HEK293T cells transfected with either wild-type CD80-CDS (CD80_Wt) or its m^6^A site mutation (CD80_Mut). Numbers below plot in (**a**, **b**) and above plot in **c** indicate the amount of transfected plasmids. **d** mRNA level of Firefly luciferase in HEK293T cells transfected with empty pMIR (Vector) or plasmids bearing wild-type (Wt) Tirap 3′-UTR (left) or CD40 3′-UTR (middle) either, or their m^6^A site mutation 3′UTR (Mut). The results were normalized by Renilla luciferase and presented relative to those transfected with a vector, set as 1. Right: mRNA level of Flag-CD80 CDS in HEK293T cells transfected with either wild-type CD80-CDS (CD80_Wt) or its m^6^A site mutation (CD80_Mut). Results were normalized by human GAPDH and presented relative to those transfected with CD80 (250 ng), set as 1. Numbers below indicate the amount of plasmids used for transfection. **e** RIP assay of m^6^A-modified Tirap, CD80, and CD40 mRNA fragments retrieved by m^6^A antibody in HEK293T transfected with wild-type or m^6^A site mutant Tirap-3′UTR, CD80-CDS, and CD40–3′UTR. Data from one representative of three independent experiments (**c**), and shown as mean ± SEM (a, b, d, e) of three determinants. **P < 0.01, ***P < 0.001, NS, not significant (Student’s *t*-test, two-tailed)

To further confirm whether the decreased translation of Tirap, CD80, and CD40 mRNA was caused by reduced m^6^A modification level, we conducted luciferase reporter and mutagenesis assays. We found that compared with mutant Tirap-3′UTR (Tirap_Mut) and mutant CD40–3′UTR (CD40_Mut) with A of the m^6^A sites substituted with G, ectopically expressed constructs bearing wild-type Tirap-3′UTR (Tirap_Wt) and wild-type CD40–3′UTR (CD40_Wt) substantially increased the luciferase activity (Fig. 6a, b) but with similar mRNA expression of Firefly luciferase (Fig. 6d). Similarly, overexpression of wild-type CDS of CD80 (CD80_Wt) resulted in higher protein levels but similar mRNA levels of Flag-CD80 compared with mutant CDS of CD80 (CD80_Mut) with a synonymous mutation with G substituted with T to disrupt the RRACH motif (Fig. 6c, d). Moreover, meRIP qPCR assays confirmed that overexpressed Tirap_Wt, CD80_Wt, and CD40_Wt had high abundance of m^6^A modification, while Tirap_Mut, CD80_Mut, and CD40_Mut contained no m^6^A modification in the mutated m^6^A sites (Fig. 6e). Taken together, Mettl3-mediated m^6^A modification promotes the translational expression of Tirap, CD80, and CD40 both in vivo and in vitro.

### m^6^A-dependent translational enhancement of CD40 and CD80 is positively associated with Ythdf1

**Fig. 7:**
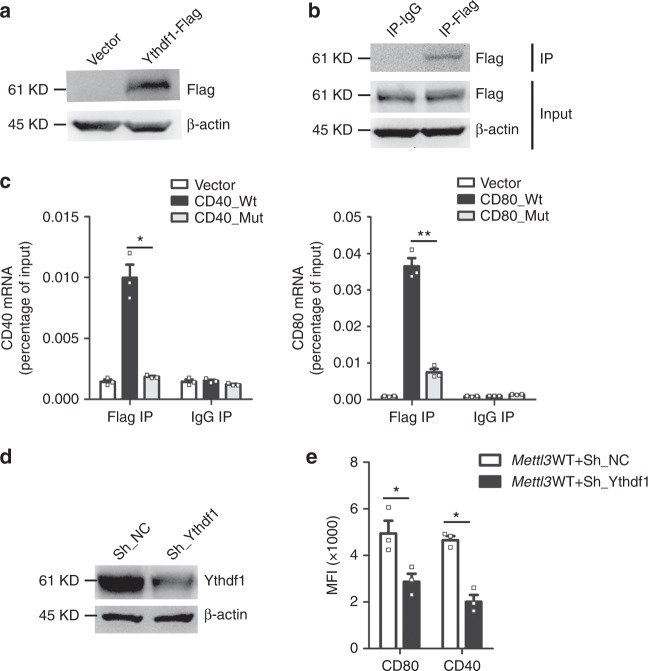
m^6^A-dependent translational enhancement of CD40 and CD80 is positively associated with Ythdf1. **a** Expression of Flag-Ythdf1 transfected into HEK293T cells. **b** Immunoprecipitation (IP) of the Flag-Ythdf1 in HEK293T cells transfected with Flag-Ythdf1 plasmid. **c** RIP-qPCR of the Flag-Ythdf1-associated RNA in HEK293T cells co-transfected with Ythdf1 and CD40_Wt or CD40_Mut (left), or in HEK293T cells co-transfected with Ythdf1 and CD80_Wt or CD80_Mut (right). **d** Expression of Ythdf1 in sorted *Mettl3*WT maDC transfected with negative control Sh_plasmid (Sh_NC) or Sh_Ythdf1. **e** Protein expression of CD40 and CD80 in *Mettl3*WT maDC transfected with negative control Sh_plasmid (Sh_NC) or Sh_Ythdf1. Data are from one representative of three independent experiments (**a**, **b**,** d**) and shown as mean ± SEM (**c**, **e**) of three determinants. *P < 0.05, **P < 0.01 and NS, not significant (Student’s *t-*test, two-tailed)

Considering the above lifetime sequencing (Fig. 4d) and ribosome-profiling data (Fig. 5d, e), we wondered whether the m^6^A reader protein Ythdf1, which had been reported to enhance translation of targeted transcripts^42,43^, was associated with the increased translation of CD40 and CD80. Immunoprecipitation (IP) experiments revealed that Ythdf1 associated with CD40_Wt or CD80_Wt mRNA more efficiently than with the CD40_Mut mRNA and CD80_Mut mRNA, respectively, indicating Ythdf1 could recognize the m^6^A-modified mRNAs we analyzed (Fig. 7a–c). Consistently, lentivirus-mediated knockdown of *Ythdf1* decreased the protein expression of CD40 and CD80 in maDC of *Mettl3*WT mice (Fig. 7d, e), but not of the m^6^A-deprived CD40 and CD80 in *Mettl3*KO mice (Supplementary Fig. 7). These data suggested a role of Ythdf1 in promoting the translation of CD40 and CD80 mRNA.

In conclusion, we demonstrate that Mettl3-mediated m^6^A of CD40, CD80, and Tirap plays an important role in promoting DC activation and maturation: the upregulated expression of CD40 and CD80 contributes to increased antigen presentation and T-cell stimulation by DC, and the higher expression of Tirap contributes to strengthened TLR4/NF-κB signaling and increased secretion of proinflammatory cytokines (Supplementary Fig. 8).

## Discussion

Here, we identified an important role of Mettl3-mediated m^6^A modification in promoting maturation and activation of DC via upregulating translation of the key transcripts in DC, including CD40, CD80, and the TLR signaling adaptor Tirap. Dysregulation of DC-mediated immune activation and tolerance is closely associated with various pathological conditions and DC vaccine represents a promising strategy for treatment of cancer and infectious diseases. The identification of Mettl3, as a positive regulator of DC function and TLR4 signaling, may have great relevance to the pathogenesis of diseases related with DC dysfunction, and may facilitate cancer immunotherapy, such as adoptive infusion of DC vaccine or design of potent cellular adjuvant.

We showed that Ythdf1 recognizes m^6^A in CD40, CD80, and Tirap transcripts and promotes their protein translation. Recent studies have uncovered new m^6^A readers and their diversified functions. YTHDF1 and YTHDF2 are the most studied readers, promoting mRNA translation by associating with translation initiation factors or mediating mRNA degradation by cooperating with RNA-processing proteins, respectively^15,16,43^. Other readers include YTHDC1 and YTHDC2, which exert important roles in affecting mRNA splicing and translocation and increasing mRNA translation efficiency^44,45^. A newly identified m^6^A reader family containing IGF2BP1, IGF2BP2, and IGF2BP3 regulates gene expression by enhancing the stability of its targets^46^. Prrc2a, another novel m^6^A reader, controls oligodendrocyte specification and myelination by stabilizing target mRNA^47^. Our lifetime-sequencing data indicated no difference of mRNA decay between *Mettl3*WT and *Mettl3*KO BMDC; therefore we focus on Ythdf1, the only reader protein that particularly regulates mRNA translation, for further investigation. However, we did not exclude the potential binding or function of other readers in the m^6^A-modified transcripts of mature DC, which require further investigations.

Increasing studies show that m^6^A displays variable functions during innate immunity and inflammation, via cooperation with distinct m^6^A writers and readers. A recent study conducted in human foreskin fibroblasts indicated that inhibition of the m^6^A writer METTL3 and reader YTHDF2, led to an increase in the induction of interferon-stimulated genes after virus infection and showed that the m^6^A of murine *Ifnb* mRNA accelerated its mRNA degradtion^48^. Another finding suggested that METTL14 depletion reduced virus reproduction and stimulated dsDNA- or HCMV-induced IFNB1 mRNA accumulation by increasing both nascent IFNB1 mRNA production and stability^49^. Consistently, our RNA-seq data revealed upregulated ISGs in LPS-stimulated *Mettl3*KO BMDC. One recent study reported that antitumor immunity was controlled through mRNA m^6^A methylation and YTHDF1 in DCs. They demonstrated that loss of YTHDF1 in classical DCs enhanced the cross-presentation of tumor antigens and the cross-priming of CD8^+^ T cells in vivo by binding to lysosomal protease transcripts and increasing their translation^42^. In our study, we showed decreased DC activation and function for promoting CD4^+^ T-cell proliferation after inhibition of m^6^A modification. The discrepancy in DC function upon m^6^A inhibition might be explained by different experimental models; however, these studies indicate an indispensable but flexible role of m^6^A in innate immune response and antitumor immunity.

In our study, MeRIP-seq data of imDC, maDC, and DCreg indicated a dynamic change of mRNA m^6^A modification, which are strongly associated with immune response, cell cycle, and DNA damage repair system. Up to now, the mechanisms of m^6^A target specificity—why m^6^A selectively appeared in certain transcripts while not in others, remain poorly understood. Previous report showed that m^6^A modification motif RRACH shared high similarity with the seed sequence of microRNA-binding sites, which hinted that microRNA may be involved in regulating mRNA m^6^A modification in a sequence-matching manner^50^. Classic theory confirmed that the m^6^A methyltransferase complex localized to nuclear speckles, which associated closely with transcription and splicing factors, participating in mRNA transcription and processing^51^. The heterodimers Smad2 and Smad3, two important downstream transcription factors of TGF-β, were found to be colocalized with the METTL3–METTL14–WTAP complex; in addition, SMAD2 and SMAD3 promoted binding of the m^6^A methyltransferase complex to a subset of transcripts involved in early cell-fate decisions^52^. Whether transcription factors, or perhaps other epigenetic enzymes could mediate the selection of m^6^A targets in different cells, is worth further investigations.

## Methods

### Mice

C57BL/6 mice were from Joint Ventures Sipper BK Experimental Animal Company (Shanghai, China). OT-II mice (which have transgenic expression of a T-cell antigen receptor specific for chicken ovalbumin amino acids 323–339 (OVA_(323–339)_) (amino acids 323–339) (ISQAVHAAHAEINEAGR) (Sigma-Aldrich) in the context of the MHC class II molecule I-Ab), CD45.1^+^ congenic mice, and transgenic CD11c-cre mice were from The Jackson Laboratory. Mice bearing a *Mettl3*^fl^ allele (*Mettl3*^fl^ mice) were from Chinese Academy of Sciences^53^. Mice lacking *Mettl3* exon2, exon3, and exon4 specifically in DC, were generated by breeding of *mettl3*^fl^ mice with CD11c-Cre mice. All mice were maintained under pathogen-free conditions and were used at 6–8 weeks of age unless indicated otherwise. All animal experiments were carried out according to the National Institute of Health Guide for the Care and Use of Laboratory Animals, with the approval of the Scientific Investigation Board of Second Military Medical University (Shanghai, China).

### BMDC preparation

Bone marrow (BM) from 6 week’s C57BL/6 mice was cultured in RPMI-1640 medium (PAA Laboratories) supplemented with 10% FBS (fetal bovine serum) (Gibco), recombinant mouse GM-CSF (10 ng/ml) (R&D), and IL-4 (1 ng/ml) (R&D). After 3 days, non-adherent cells were gently removed and the remaining cells were further cultured with fresh medium containing GM-CSF and IL-4. On the fifth day, loosely adherent cells were subjected to positive selection with magnetic beads coated with anti-mouse CD11c and were defined as immature DC (imDC). The CD11c^+^ imDC stimulated with LPS (100 ng/ml) for 24 h was defined as mature DC (maDC). maDC cocultured with spleen endothelial cells in RPMI-1640 supplemented with 5% FBS for 7 days was defined as regulatory DC (DCreg)^32^.

### Analysis of m^6^A/A ratio using HPLC–MS/MS

Total RNA of DC was isolated with Trizol reagent (Invitrogen), and mRNA was enriched by Dynabeads mRNA Purification Kit (Invitrogen). Removal of ribosomal RNA was confirmed by 2200 Tape Station detection (Agilent). In total, 2.5 μl of 10x Reaction Buffer (20 mM of ZnCl_2_, 100 mM of NaCl) and 1 μl of Nuclease P1 (1.2 U/μl) (Sigma) were added to 350 ng of purified mRNA and incubated at 37 ℃ for 2 h after adding H_2_O to a total volume of 2 μl. Then 2.5 μl of CIAP Buffer and 1 μl of CIAP (Promega) were added and incubated at 37 ℃ for another 2 h. The mix was diluted with H_2_O to 100 μl and filtered through a 0.22-µm filter (4 mm in diameter) (Nalgene) and then loaded to a C18 reverse-phase column coupled online to Agilent 6410 QQQ triple-quadrupole LC mass spectrometer in positive electrospray ionization mode. The nucleosides were quantified using the nucleoside to base on mass transitions of 268–136 (A), and 282–150 (m^6^A). A standard curve was obtained from pure nucleoside standards running at the same batch of samples. The m^6^A/A ratio in poly(A) RNA was quantified based on the calculated concentrations^13^.

### Dot-blot assay

Total RNA of BMDCs was isolated with Trizol reagent (Invitrogen) and mRNA was enriched using Dynabeads mRNA Purification Kit (Invitrogen). The quality and quantity were monitored by 2200 Tape Station (Agilent). mRNA in a volume of 1.5 μl was denatured by heating at 72 °C for 5 min, followed by chilling on ice immediately. Then, mRNA was spotted in duplicate on Biodyne Nylon Transfer Membranes (Pall) and cross-linked to the membrane by UV using HL-2000 HybriLinker (UVP). One of the membranes was blocked with 5% BSA in TBST and the m^6^A level was detected using the m^6^A-specific antibody (Synaptic Systems, 202003, 1:1000), the other membrane was methylene blue stained as loading control.

### m^6^A-meRIP-Seq and m^6^A-meRIP qPCR

BM from about 20 mice was induced into imDC or DCreg, while BM from 12 mice was induced into maDC as mentioned above. Total RNA was isolated with Trizol reagent (Invitrogen) and mRNA was enriched by Dynabeads mRNA Purification Kit (Invitrogen). Removal of ribosomal RNA was confirmed by 2200 Tape Station detection (Agilent). About 8 μg of mRNA was fragmented to 100–200 bp in length using fragmentation reagent (Ambion) and 100 ng was separated as input. The remaining fragmented RNA was mixed with 50 μl of Dynabeads Protein A (Life Technology) pre-mixed with 16 μg of anti-m^6^A antibody overnight in IP buffer (150 mM NaCl, 10 mM Tris-HCL, and 0.1% NP-40 supplemented with RNase inhibitor and protein inhibitor). The beads–antibody–RNA mix was washed with high-salt washing buffer twice, middle-salt washing buffer twice, and low-salt washing buffer twice separately. Following the last wash, 500 μl of Trizol was added to the mix to extract the binding RNA. Both input and m^6^A IP samples were prepared for the next-generation sequencing (NGS) by the Ribobio (China). The NGS library preparation was constructed by NEBNext Ultra RNA Library Prep Kit for Illumina.

For the identification and analysis of the m^6^A peaks, raw reads were aligned to the reference genome (mm10) using TopHat (v2.0.14)^54^ after removing the adaptor. RPKM (reads per kilobase per million mapped reads) was calculated by Cuffnorm^55^. For m^6^A Seq, the same method was used^56^. Briefly, the longest isoform was used if multiple isoforms were detected. Aligned reads were extended to 150 bp (average fragment size) and converted from genome-based coordinates to isoform-based coordinates in order to eliminate the interference from an intron in peak calling. To call m^6^A peaks, the longest isoform of each mouse gene was scanned using a 100-bp sliding window with 10-bp steps. To reduce bias from potential inaccurate gene structure annotation and the arbitrary usage of the longest isoform, windows with reads counts less than 1/20 of the top window in both m^6^A IP and input sample were excluded. For each gene, the reads count in each window was normalized by the median count of all windows of that gene. A negative binomial model was used to identify the differential windows between IP and input samples by using the edgeR package^57^. The window was called as positive if FDR < 1% and log_2_(enrichment score) ≥ 1 in both replicates. Overlapping positive windows were merged. The following four numbers were calculated to obtain the enrichment score of each peak (or window): read count of the IP sample in the current peak/window (a), median read count of the IP sample in all 100-bp windows on the current mRNA (b), read count of the input sample in the current peak/window (c), and median read count of the input sample in all 100-bp windows on the current mRNA (d), the enrichment score of each window was calculated as (a) × (d)/(b) × (c). DAVID tool was used to perform enrichment analysis^58^.

For m^6^A-meRIP-qPCR, we started with ~2 μg of total RNA of CD11c^+^ BMDC from *Mettl3*WT and *Mettl3*KO mice. Using the same protocol with scale-down reagents, the IP-extracted RNA together with the input-extracted RNA was resolved in 10 μl of RNase-free water. m^6^A enrichment was analyzed on light Cycler 480 (Roche Diagnostics) with indicated primers. Socs1 with m^6^A-modified transcript both in DC and T cells was used as positive control. Ctla4 with no positive m^6^A-modified transcript in our MeRIP data was used as negative control.

### Plasmid construction and mutagenesis assays

Recombinant vectors encoding mouse Mettl3 (National Center for Biotechnology Information (NCBI) reference sequence NM_019721) and CD80 (NCBI reference sequence NM_009855) were constructed by PCR-based amplification from cDNA of mouse BMDC and then subcloned into the pcDNA3.1 eukaryotic expression vector (Invitrogen). The Tirap-3′UTR (NCBI reference sequence NM_001177845) and CD40–3′UTR (NCBI reference sequence NM_011611) were amplified by PCR from the cDNA of the mouse BMDC and then inserted into downstream of firefly luciferase of pMIR-REPORT vector (Luciferase miRNA Expression Reporter Vector, Ambion). Mutagenesis assays were performed by the KOD Plus Mutagenesis Kit (Toyobo). The primers used for plasmid construction were shown in Supplementary Table 2. For plasmid construction, we have inserted the whole nucleotide of Tirap-3′UTR or CD40–3′UTR or CD80-CDS into pMIR or pcDNA3.1 vectors. Tirap_mut, CD80_mut, and CD40_mut had disrupted the m^6^A RRACH motif with the highest enrichment score (Supplementary Table 3). Wild types or mutations of the specific m^6^A modification peak of the three mRNA are listed in Supplementary Table 4. All constructs were confirmed by DNA sequencing.

### Lentiviral plasmid construction and transfection

The cDNA encoding Flag-tagged Mettl3 (NCBI reference sequence NM_019721) was amplified from the corresponding plasmid by PCR and then subcloned into the GV365 plasmid (Ubi-MCS-3FLAG-CMV-EGFP) (Genechem). The GV365 plasmids were then co-transfected into HEK293 cells with the lentiviral genomic plasmids. The recombinant lentiviruses were amplified, purified, and stored according to the Lentivirus Vector Construction Manual (GeneChem). For lentivirus transfection, CD11c^+^ BMDC was transfected with a lentivirus (multiplicity of transfection (MOI), 10:1) encoding wild-type or mutant Mettl3 or with negative control lentivirus and then were cultured for 72 h. The surface markers of GFP and CD11c double-positive cells (about 50% of CD11c^+^ cells) were analyzed. Ythdf1 shRNA lentiviral plasmids were constructed according to the same protocol using GV248 plasmid (hU6-MCS-Ubiquitin-EGFP-IRES-puromycin), the target sequence of Ythdf1 was ACAACAAACCTGTCACAAA, and the oligo synthesis information was listed in Supplementary Table 5.

### Flow cytometry

The phenotypes of DC as well as the proliferation assay of CFSE-labeled T cells were determined by flow cytometry^35^. For cell surface staining, single-cell suspensions were incubated for 15 min at 4 °C with PE Hamster anti-mouse CD11c (0.4 μg/ml) (BD Biosciences, 553802), PerCP-Cy5.5 Hamster anti-mouse CD80 (0.2 μg/ml) (BD Biosciences, 560526), PerCP-Cy5.5 anti-mouse CD86 (0.2 μg/ml) (Biolegend, 105027), PE-Cy7 anti-mouse CD40 (0.1 μg/ml) (Biolegend, 124621), PE-Cy7 anti-mouse I-Ab (0.1 μg/ml) (Biolegend, 116420), PE-Cy7 anti-mouse CD4 (0.2 μg/ml) (Biolegend, 100422), APC anti-mouse TCR Vβ5.1 antibody (0.2 μg/ml) (Biolegend, 139511), and FITC anti-mouse CD45.2 antibody (0.2 μg/ml) (Biolegend, 109805). Samples were washed and analyzed by FACS versus flow cytometry (BD Biosciences), and the gating strategies were shown in Supplementary Fig. 9.

### Ribosome profiling and qPCR of ribosome-associated mRNA

The procedure was adapted from the previous report^16^. BMDC from six pairs of *Mettl3*WT mice and littermate *Mettl3*KO mice was prepared. Before collection, cycloheximide (CHX) was added to the media at 100 mg/ml for 7 min. The media was removed, and the cells were collected with 5 ml of cold PBS with CHX (100 mg/ml). The cell suspension was spun at 600 g for 5 min and the cell pellet was washed once by 1 ml of PBS–CHX. One milliliter of lysis buffer (10 mM Tris, pH 7.4, 150 mM KCl, 5 mM MgCl_2_, 100 mg/ml CHX, 0.5% Triton X-100, freshly added 1:100 protease inhibitor, and 40 U/ml recombinant RNase inhibitor (RRI)) was added to suspend the cells and then kept on ice for 15 min with occasional pipetting and rotating. After centrifugation at 15,000 g for 15 min, 8 μl of DNase RQ1 (Promega) was added to the lysate. The lysate was then split by the ratio of 1:5 (Portion I/Portion II). Three microliters of RRI was added to Portion I, 80 μl of MNase buffer, and 3.5 μl of MNase (7,000 gelunits, BioLabs) was added to Portion II. Both portions were kept at room temperature for 15 min, and then 8 μl of RRI was added to Portion II to stop the reaction. Portion I was saved and mixed with 500 μl of TRIzol to purify input mRNA. Portion II was used for ribosome profiling.

In total, 15/45% w/v sucrose gradient was prepared in a lysis buffer without Triton X-100. Portion II was loaded onto the sucrose gradient and centrifuged at 4 °C for 3.5 h at 288244 g (Beckman, rotor SW41). The sample was then fractioned and analyzed by Gradient Station (BioCamp) equipped with UV monitor (BioRad) and fraction collector. The fractions corresponding to 80S monosome (not 40S or 60S) were collected, combined, and mixed with an equal volume of TRIzol to purify the RNA. The RNA pellet was dissolved in 15 μl of water, mixed with 15 μl of TBE-urea-loading buffer (Invitrogen), and separated on a 10% TBE-urea gel. A 21-nt and a 42-nt ssRNA oligo were used as size markers, and the gel band between 21 nt and 42 nt was cut. The RNA of the gel was extracted using Poly-gel RNA extraction kit (Promega). RNA was concentrated by ethanol precipitation and finally dissolved in 10 μl of RNase-free water.

Input mRNA: the input RNA was first purified by TRIzol and the input mRNA was then separated by Dynabeads mRNA Purification Kit (Invitrogen). The resulting mRNA was concentrated by ethanol precipitation and dissolved in 10 μl of RNase-free water. The mRNA was fragmented by RNA fragmentation kit (Ambion). Library construction: the end structures of the RNA fragments of ribosome profiling and mRNA input were repaired by T4 PNK: (1) 3′ dephosphorylation: RNA (20 μl) was mixed with 2.5 μl of PNK buffer and 1 μl of T4 PNK, and kept at 37 °C for 1 h; (2) 5′-phosphorylation: to the reaction mixture, 1 μl of 10 mM ATP and 1 μl extra T4 PNK were added, and the mixture was kept at 37 ℃ for 30 min. The RNA was purified by 500 ml of TRIzol reagent, and finally dissolved in 10 ml of water. The library was constructed by Tru-seq small RNA sample preparation kit (Illumina). The sequencing data obtained from ribosome profiling (portion II) were denoted as ribosome-protected fragments and those from RNA input (portion I) as mRNA input. Translation efficiency was defined as the ratio of ribosome-protected fragments and mRNA input, which reflected the relative occupancy of 80S ribosome per mRNA species.

For qPCR analysis, the same protocol as ribosome profiling was used but without MNase treatment. The 80S monosome- associated RNA and input RNA from *Mettl3*WT and *Mettl3*KO mice were extracted by Trizol and dissolved in 50 μl of RNase-free water. After quantification by nanodrop, the same amount of RNA was used for RT-qPCR to compute the relative translation efficiency of indicated genes. Itgam, encoding CD11b as a conserved phenotypic marker of BMDC, was used as negative control in the ribosome profiling.

### RNA-seq and RNA-seq for mRNA lifetime

For RNA-seq, we used two pairs of wild-type and littermate knockout mice for the experiment. Total RNAs from LPS-stimulated BMDCs were isolated with TRIzol and mRNA was then separated by Dynabeads mRNA Purification Kit (Invitrogen). Standard illumine HiSeq2000 sequencing was applied in Sequencing. Raw RNA-sequencing reads were aligned to the mouse genome (mm10) with Tophat. Genes were considered significantly differentially expressed if showing ≥ 1.5-fold change and < 0.01 *P* value. Gene GO enrichment analysis and enriched KEGG pathways were obtained through online bioinformatics tools. Q-value was calculated as a correction of *p*-value using Audics.

For mRNA lifetime, we used two pairs of wild-type and littermate knockout mice for the lifetime experiment. The procedure was adapted from the previous report^15^. Briefly, actinomycinD was added to 5 μg/ml at 6, 3, and 0 h before collection. The total RNA was purified by TRIzol reagent. Before construction of the library with Tru-seq mRNA sample preparation kit (Illumina), ERCC RNA spike-in control (Ambion) was added to each sample proportional to the total RNA according to the instructions. The degradation rate of RNA k was estimated by

$${\mathrm{Log}}_2\left( {A_t/A_0} \right) = - kt$$

where *t* is transcription inhibition time (h), *A*_*t*_ and *A*_0_ represent mRNA quantity (attomole) at time t and time 0. Two *k* values were calculated: time 3 h versus time 0 h, and time 6 h versus time 0 h. The final lifetime was calculated by using the average of *k*_3h_ and *k*_6h_.

$$t_{1/2} = 2/k_{3{\mathrm{h}}} + k_{6{\mathrm{h}}}$$

### Dual-luciferase report

According to published research, the pMIR vector was used to investigate the function of m^6^A located at 3′UTR. For dual-luciferase reporter assay, 300 or 400 ng of wild-type or mutant CD40–3′UTR (or Tirap-3′UTR) and 75 or 100 ng of pRL-TK (renilla luciferase control reporter vector) were co-transfected into HEK293T cells in a 24-well plate. The relative luciferase activities were accessed 48 h post transfection by Dual-Luciferase Reporter Assay System (Promega). Each group was repeated in triplicate.

### RNA immunoprecipitation (RIP)

Cells were washed twice with ice-cold PBS and ruptured with CLB buffer (Cell Signaling Technology) with cocktail protein inhibitor and the RNase inhibitor. After lysis, the RNA in the whole-cell lysis was fragmented into ~500 bp in length by the ultrasonic sound (1/10 volume was separated as input) and then immunoprecipitation was carried out with m^6^A, Flag, or IgG antibodies overnight at 4 °C. The precipitated RNA was extracted using Trizol reagent and was reverse transcribed with a PrimeScript RT-PCR Kit. qPCR analysis of the retrotranscribed RNA was performed with specific primers as indicated.

### RT-PCR and qPCR

Total RNA was extracted from cultured cells with TRIzol reagent (Invitrogen). First-strand cDNA was synthesized with a PrimeScript RT-PCR Kit. The relative expression of mRNA or the RNA fragments precipitated in RIP assays was quantified by real-time PCR with SYBR Premix ExTaq kit and was normalized to the expression of β-actin or the IgG control. cDNA was amplified on the Light Cycler (Roche Diagnostics). The 2^−ΔΔCt^ change-in-cycling-threshold method was used for calculation of relative changes in expression. Sequences of the primers for qRT-PCR are shown in Supplementary Table 6.

### Immunoblotting

Cells were washed twice with ice-cold PBS and ruptured with CLB buffer (Cell Signaling Technology) containing PMSF and cocktail inhibitor. Cell lysates were resolved by SDS-PAGE and transferred onto nitrocellulose membranes and then blotted. Specific antibodies used are listed below: anti-Mettl3 (15073–1-AP, 1:1000) antibody was from Proteintech. Anti-Mettl14 antibody (HPA038002, 1:000) was from Sigma; Anti-Fto (ab124892, 1:1000), anti-Wtap (ab118339, 1:500) were from Abcam; antibody to p65 phosphorylated at Ser536 (3031S, 1:1000), antibody to IKKα-IKKβ phosphorylated at Ser176 and Ser180 (2697S, 1:1000), antibody to Erk phosphorylated at Thr202 and Tyr204 (9106S, 1:1000), antibody to Jnk phosphorylated at Thr183 and Tyr185 (9255S, 1:1000), anti-β-actin (3700S, 1:10000), anti-Flag-HRP (2044S, 1:2000), anti-p65 (6956S, 1:1000), anti-p38 (9212S, 1:1000), anti-IκBα (9242S, 1:1000), and anti-IKKβ (8943S, 1:1000) were from Cell Signaling Technology. All of the unprocessed scans of the blots were shown in the Source Data file.

### Listeria infection

In total, 2 × 10^5^ pfu wide-type *Listeria monocytogens* (LM) was tail intravenously injected into *Mettl3*WT mice or littermate *Mettl3*KO mice. Three days later, the total spleen was collected and a single-cell suspension was harvested for flow cytometry analysis.

### Mixed lymphocyte reactions (MLR)

Splenic CD4^+^ T lymphocytes were purified from OT-II mice using anti-CD4 microbeads (Miltenyi Biotech). LPS-activated maDC was pulsed for 2 h with OVA_(323–339)_ (200 nM) or left unpulsed as control and then was cultured at a ratio of 1:10 (DC/T cell) with OT-II CD4^+^ T cells (labeled with CFSE). Four days later, T-cell proliferation was measured by flow cytometry as the dilution of CFSE and the supernatants were used for cytokine detection in ELISA^35^.

### Immunization protocol for in vivo experiment

For an assay of antigen-specific T-cell responses in vivo, naive OT-II CD4^+^ T cells were purified from the spleen of OT-II mice and adoptively transferred (1 × 10^6^ cells per mouse) into CD45.1^+^ mice by tail intravenous injection. After 1 day, mature or lentiviral plasmids transfected with BMDCs from *Mettl3*WT or *Mettl3*KO mice were pulsed for 2 h with OVA_(323–339)_(100 μg/ml), or left unpulsed, as controls were transferred subcutaneously into the hind footpads of recipient mice (3 × 10^5^ cells per mouse). Four days later, draining popliteal lymph nodes were collected, and the proportion of CD45.2^+^ T cells was analyzed^35^.

### Statistical analysis

Statistical significance was assessed by Student’s *t-*test, with a value of *P* < 0.05 considered statistically significant. The statistical tests were justified as being appropriate according to assessment of normality and variance of the distribution of the data. No randomization or exclusion of data points was used. No “blinding” of investigators was done. Sample sizes were chosen according to previous experience and preliminary studies to ensure adequate power.

### Reporting summary

Further information on research design is available in the Nature Research Reporting Summary linked to this article.

## Supplementary information

Supplementary information

Peer Review File

Reporting Summary

Source Data

## Data Availability

The source data that support the findings of this study are available from the corresponding author upon request. The sequencing data for meRIP, mRNA, lifetime, and ribosome profile have been deposited in the Gene Expression Omnibus under accession number GSE108333. The source data of an immunoblot underlying Figs. 1a, 1b, 2e, 4d, 6c, 7a, 7b, 7d and Supplementary Fig. 2b are provided as a Source Data file.
