# Supplementary material for: Mettl3-mediated mRNA m6A methylation promotes dendritic cell activation
Source: Nat Commun. 2019 Apr 23;10:1898. doi: 10.1038/s41467-019-09903-6 (PMC6478715; doi:10.1038/s41467-019-09903-6)
Supplement: Supplementary file 1 — Supplementary information [file 41467_2019_9903_MOESM1_ESM.pdf]

SUPPLEMENTARY INFORMATION FOR

Mettl3-mediated mRNA m^6A methylation promotes dendritic cell activation

Huamin Wang et al

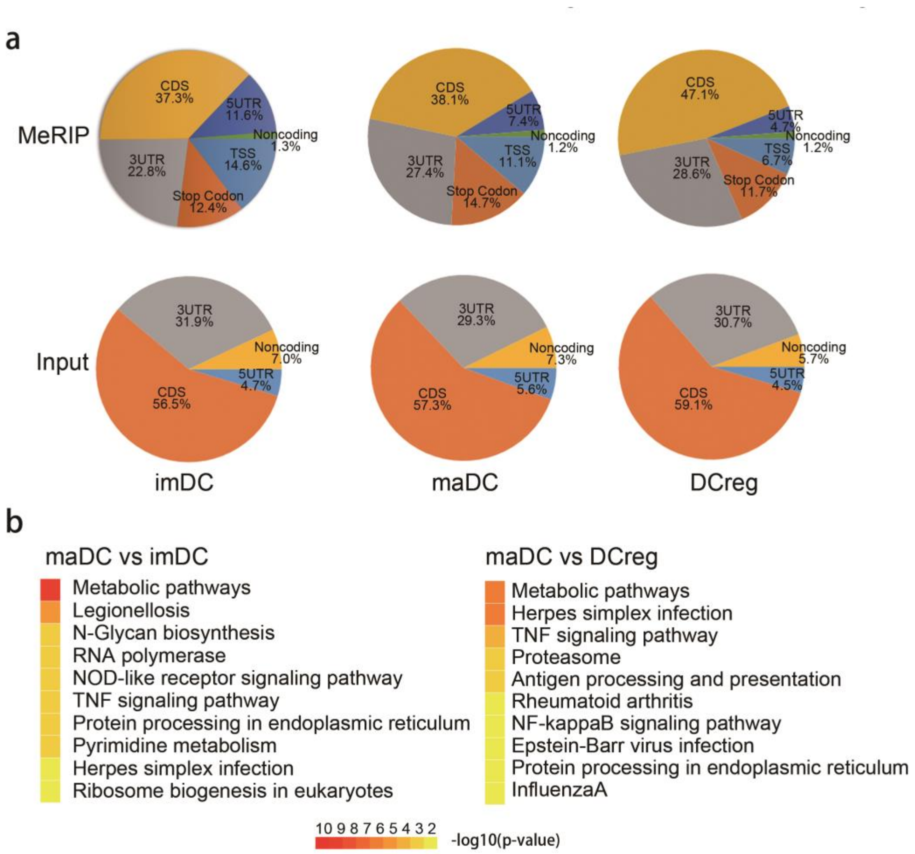

Supplementary Figure 1 The distribution of m^{6}A peaks in imDC, maDC and DCreg.

a Transcriptome-wide distribution of m^{6}A peaks, pie charts showing the percentage of m^{6}A peaks (up) and non-IP sample reads (down) in imDC, maDC and DCreg. **b** KEGG pathway analysis of specific m^{6}A-modified transcripts in maDC compared with imDC (left) or DCreg (right).

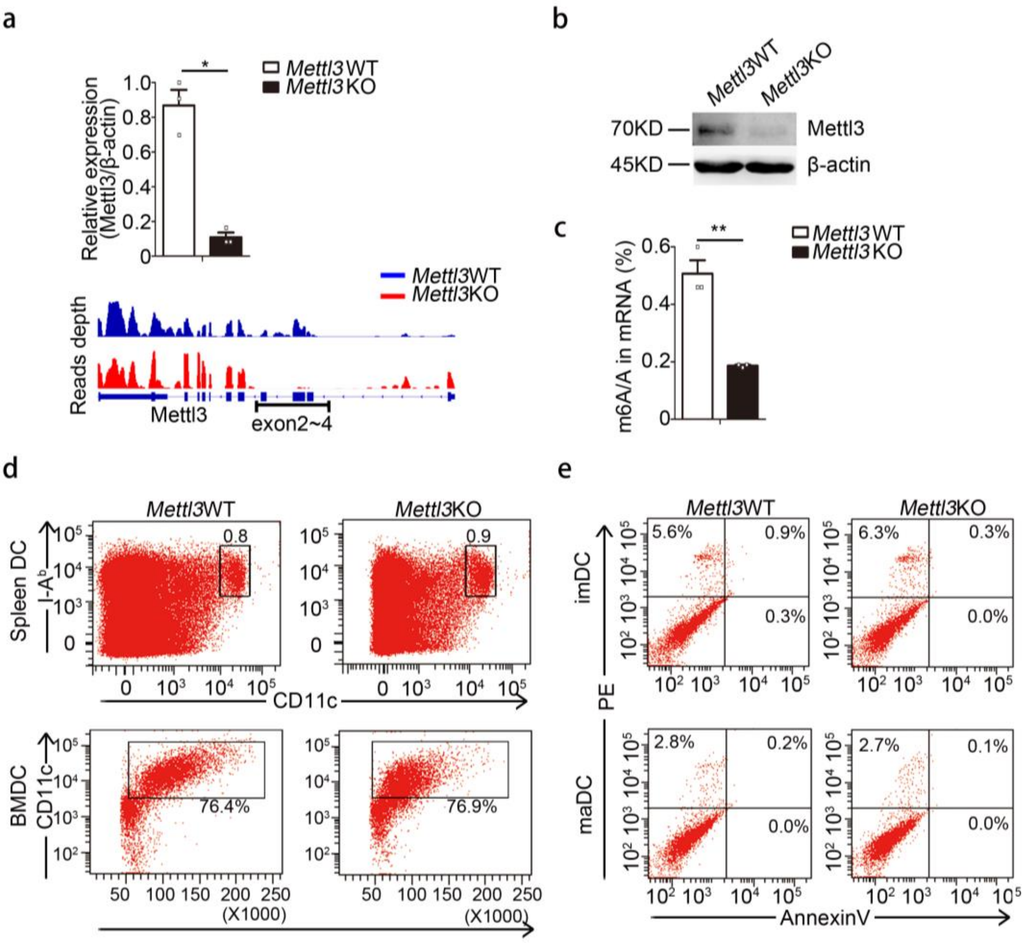

Supplementary Figure 2 *Mettl3* deficiency decreases m^{6}A level in DC and has no effect on splenic DC and BMDC generation.

a,b Mettl3 expression in *Mettl3*KO maDC was greatly impaired at mRNA level (a) and protein level (b). Results were normalized by mouse β-actin and presented relative to those of mature *Mettl3*WT maDC, set as 1. RNA-Seq data showed the exons 2~4 of Mettl3 were successfully removed in Mettl3KO DCs (a, down). **c** m^{6}A level in *Mettl3*KO maDC detected by HPLC-MS/MS is decreased to 1/3 of that in *Mettl3*WT maDC. **d** The rate of CD11c positive cells in splenocytes or BMDC of *Mettl3*WT and *Mettl3*KO mice. **e** The apoptotic level of immature *Mettl3*WT DC and

*Mettl3*KO DC(up), of mature *Mettl3*WT DC and *Mettl3*KO DC(down). Data are from one representative of three independent experiments (b, d, e) and shown as mean±SEM (up panel of a, c) of three determinants. *P < 0.05 **P < 0.01 and NS, not significant (Student's t-test, two-tailed).

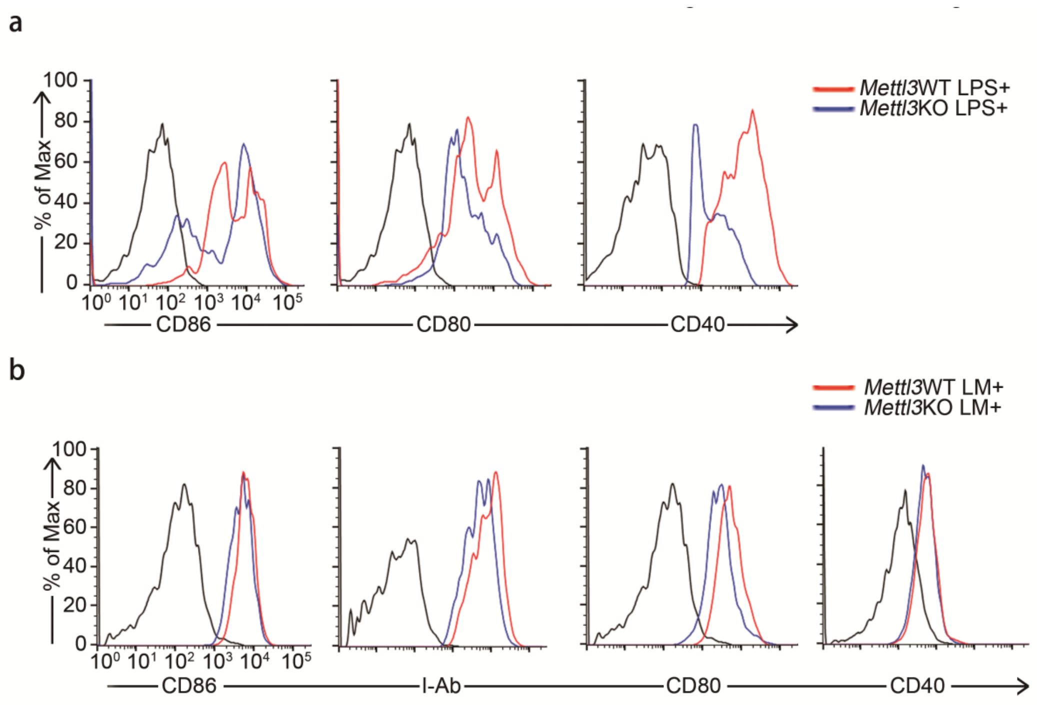

Supplementary Figure 3 ***Mettl3*** **deficiency impairs the expression of phenotypic molecules of splenic DC** ***in vivo*****.**

a Indicated expression of CD86, CD80 and CD40 of LPS stimulated *Mettl3*WT and *Mettl3*KO splenic DC. **b** Expression of indicated molecules of splenic DC from *Mettl3*WT and *Mettl3*KO mice 3 days after LM infection. Data are from one representative of three independent experiments.

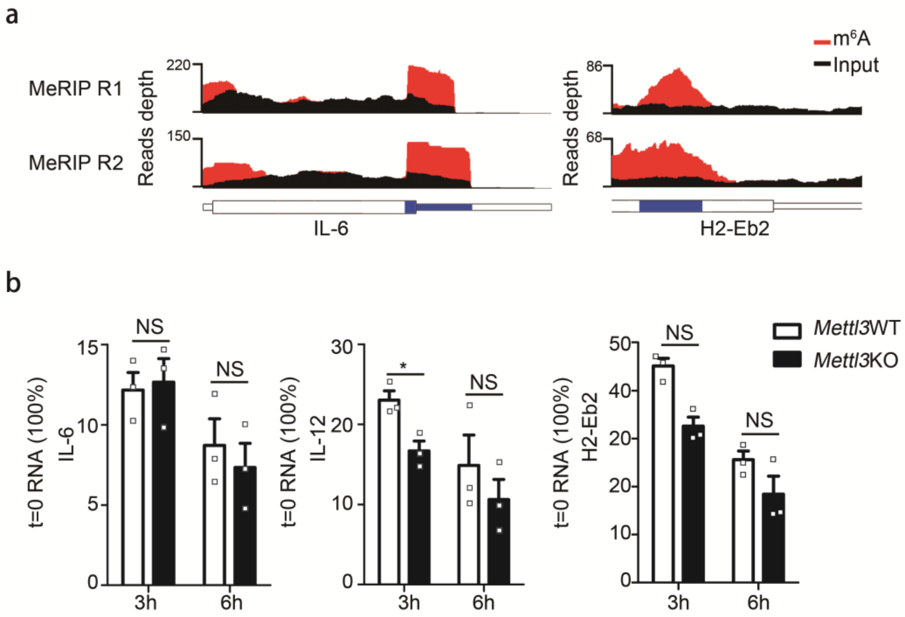

Supplementary Figure 4 No difference in the degradation level of IL-6, IL-12 and H2-Eb2 mRNA between *Mettl3*WT and *Mettl3*KO DC.

a m^6A peaks were enriched in the 3'UTRs of IL-6 and H2-Eb2 genes from m^6A RIP-seq data. **b** RNA degradation assay showed that IL-6, IL-12 and H2-Eb2 mRNA degrade equally in *Mettl3*KO maDC with that in *Mettl3*WT maDC three or six hours after actinomycin-D treatment. The residual RNAs were normalized to t = 0 in maDC. Data are shown as mean±SEM (b) of three determinants. *P < 0.05 and NS, not significant (Student's t-test, two-tailed).

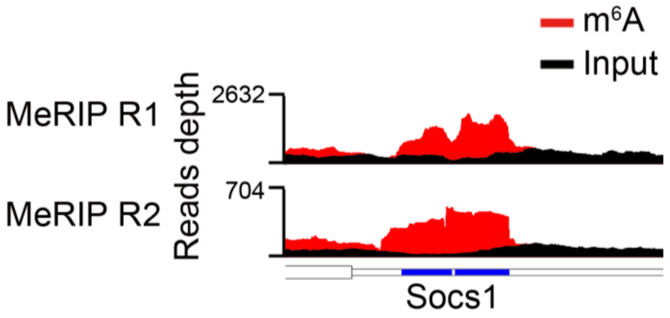

Supplementary Figure 5 m^{6}A peaks are enriched in the 3'UTR of Socs1 mRNA from m^{6}A RIP-seq data.

m^{6}A peaks marked in blue are enriched in the 3'UTRs of Socs1 gene from m^{6}A RIP-seq data in maDC. R1 and R2 was representative of meRIP replicate1 and replicate2

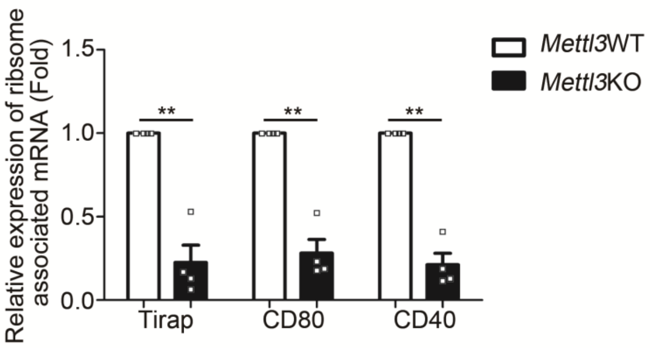

Supplementary Figure 6 *Mettl3* deficiency decreases the ribosome associated Tirap, CD80 and CD40 mRNA in maDC.

RT-qPCR of the same amount of RNA extracted from 80S monosome fraction of *Mettl3*WT and *Mettl3*KO maDC lysates. The result confirmed the decreased translation efficiency of Tirap, CD80 and CD40 in *Mettl3*KO maDC. Data are shown as mean±SEM of four determinants. **P < 0.01 (Student's t-test, two-tailed).

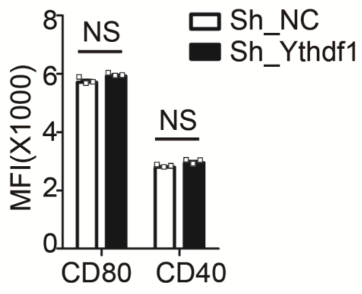

Supplementary Figure 7 Konckdown of Ythdf1 did not affect CD40 and CD80 expression in *Mettl3*KO maDC.

Protein expression of CD40 and CD80 in *Mettl3*KO maDC transfected with negative control Sh_plasmid (Sh_NC) or Sh_Ythdf1. Data are shown as mean±SEM of three determinants. NS, not significant (Student's t-test, two-tailed).

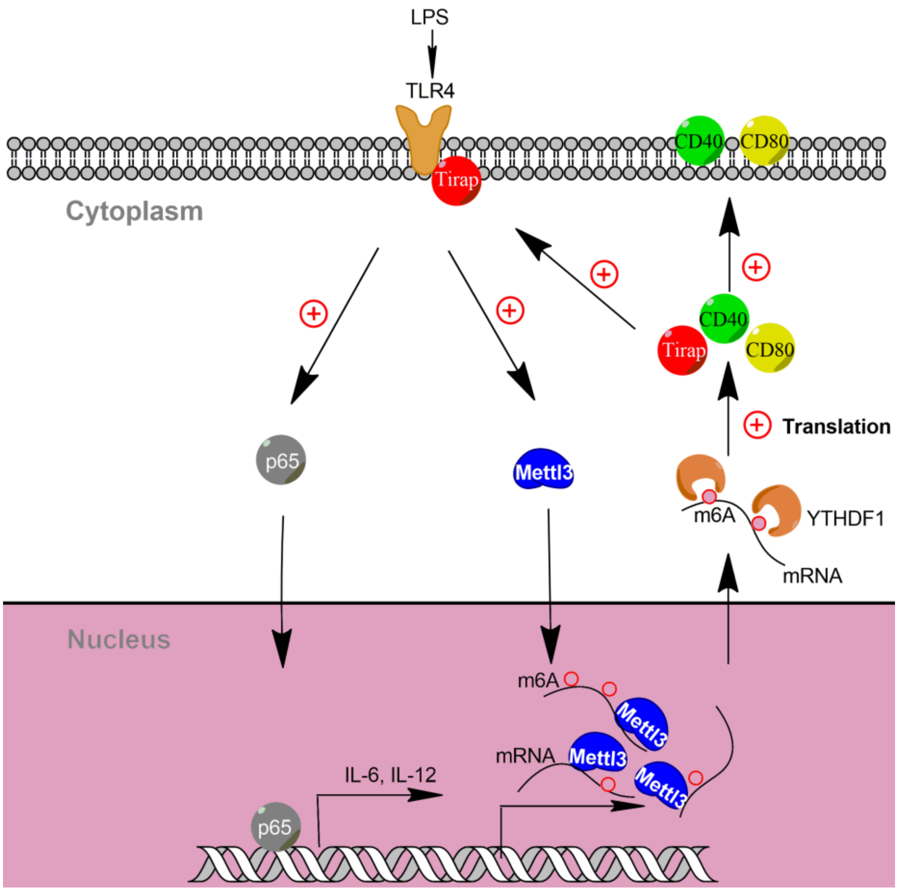

Supplementary Figure 8 The schematic model of Mettl3 in promoting DC activation via m^{6}A modification of immune transcripts.

Mettl3 mediated m^{6}A modification of the key transcripts in DC including CD40, CD80 and TLR signaling adaptor Tirap, leading to enhanced translation of CD40, CD80 and Tirap via Ythdf1. Upregulation of CD40 and CD80 in DC contributes to increased DC-mediated antigen-presentation and T cell activation, and higher expression of Tirap contributes to strengthened TLR4/NF-κB signaling and increased secretion of proinflammatory cytokines including IL-6 and IL-12, which coordinately promote DC maturation and activation.

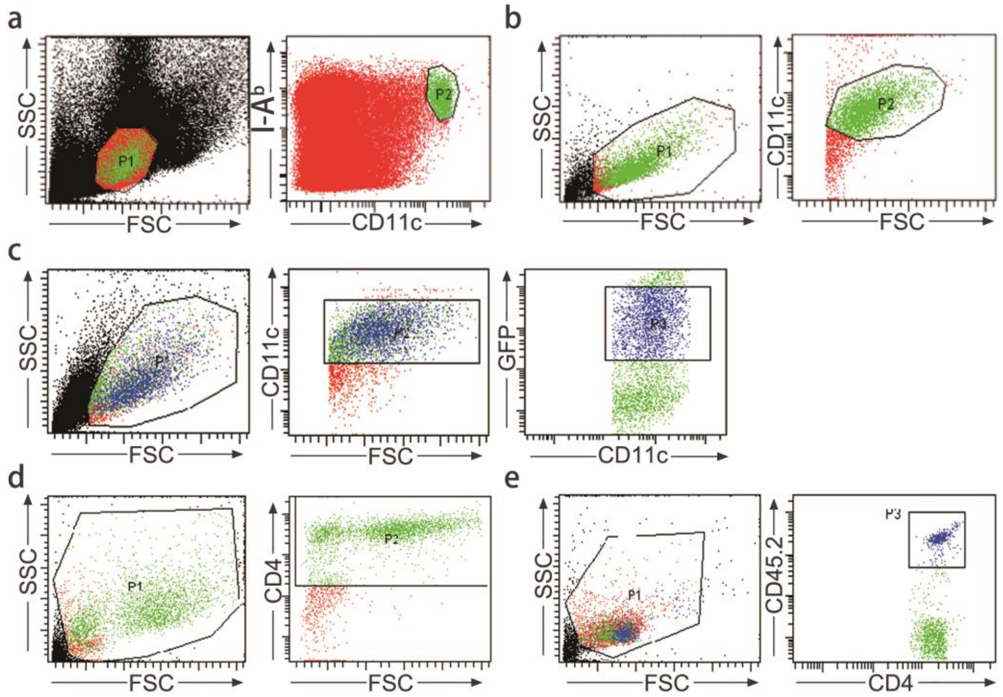

Supplementary Figure 9 The gating and sorting strategies of indicated cell subsets.

a The representative FACS plots show the gating strategies of CD11c^{+} and I-A^{b+} dendritic cell subsets from the splenocytes of mice, corresponding to Fig. 2a; Supplementary Fig. 2d up and Supplementary Fig. 3a, b. **b** The representative FACS plots show the gating strategies of CD11c^{+} dendritic cell subsets from BM stimulated with IL-4 and GM-CSF for 5~7 days, corresponding to Fig. 2c; Supplementary Fig. 2d down and Supplementary Fig. 2e. **c** The representative FACS plots show the gating strategies of lentivirus transfected CD11c^{+} dendritic cell subsets from BMDC, corresponding to Fig. 2f; Fig. 7e and Supplementary Fig. 7. **d** The representative FACS plots show the gating strategies of CD4^{+} T cell subsets from MLR cell suspension, corresponding to Fig. 3a. **e** The representative FACS plots show the gating strategies of CD4^{+} and CD45.2^{+} T cell subsets from draining popliteal lymph nodes of mice, corresponding to Fig. 3c.

Supplementary Table 1 The genes with more than 1.5 fold mRNA expression in *Mettl3*WT DC compared with *Mettl3*KO DC in both two independent RNA-seq data

The decreased gene list in *Mettl3*KO DC:
Acpp;Acss1;Akr1b8;Amz1;Ankrd24;Asb2;Atp2a3;Atp6v0c;AV051173;BC035044;Cd70;Cxcl1;Cxcl5;Dpp4;Ephx1;Fads2;Gm6377;Gsta3;**H2-Eb2**;Idi1;**Il12b**;Il1f9;**Il6**;Kctd14;Lad1;Layn;Lif;Lpin1;Me1;Mfap1b;Mlh1;Mmd;Mpc1;Mt3;Nqo1;Odc1;P2ry1;Pafah2;Panx1;Pcx;Pdzk1ip1;Pla2g4f;Plxna1;Ppbp;Ptgs2;Pvr;Sfn;Sh2d5;Slc5a2;Slpi;Spic;Tfrc;Treml4;Txnrd1

Supplementary Table 2 Primers used for cloning indicated overexpressing vectors

Primers	Sequence (5'-3')
Tirap_F(3UTR)(WT)	GCGAGCTCTGACACTTGGGCTTTCATAAGAAAAG
Tirap_R(3UTR)(WT)	GCACGCGTAGATAATTCAGCACACTATTTAATGGTA
Tirap_F(3UTR)(Mut)	GGGCTGTGCTATCGGGAAGGTCAGC
Tirap_R(3UTR)(Mut)	CTTGACAACTCGAATTTCTGACTTC
Mettl3_F(WT)	CGGAATTCATGTCGGACACGTGGAGCT
Mettl3_R(WT)	GGGGTACCCTTAAATTCTTAGGTTTAGAGATGAT
Mettl3_F(MUT)	GATGGCTGCCCCACCTGCGGATATT
Mettl3_R(MUT)	ACAACTGCAAATTTGCCCAAGATAC
CD80_F(WT)	CCCTCGAGATGGCTTGCAATTGTCAGTTG
CD80_R(WT)	CCGGTACCGAAAGGAAGACGGTCTGTTCAG
CD80_F(MUT)	GTACTTTATATGACAACACTACCTA
CD80_R(MUT)	GGTTCTTATACTCGGGCCACACTTT
CD40_F(WT)	CCACGCGTACCCTGGAACTGCTTTGGA
CD40_R(WT)	CCAAGCTTTTATTGTAAATATAAAGGTTGAGT
CD40_F(MUT)	GGCTGCTTGCTGACCTTTGAAGTTT
CD40_R(MUT)	CTCAGAGCCAGGCCCCTGCTCCCGA

Supplementary Table 3 m^{6}A modification peaks in CD40, CD80 and Tirap mRNAs in maDC

molecules	log2(enrichment) in replicate1	log2(enrichment) in replicate2	position in mRNA	RRACH motif
CD40_peak 1*	4.843527	4.490199	883~1049	GGACT
CD40_peak 2	3.004854	2.157425	1310~1481	GGACT
CD80_peak1	2.776768	2.634679	1194~1293	none
CD80_peak2	3.44684	2.520716	1294~1469	none
CD80_peak3*	1.415135	1.775139	380~554	GGACT
Tirap_peak1	1.050937	2.405992	708~889	GGACT
Tirap_peak2*	1.432959	2.241008	1461~1660	GGACT
Tirap_peak3	1.152558	1.494765	1831~1970	none
Tirap_peak 4	1.819983	1.054448	2431~2594	none

* Highest enriched m^{6}A RRACH motif

Supplementary Table 4 Wild-type or mutant m^{6}A RRACH motif in CD40, CD80 and Tirap mRNAs in maDC

molecues	m^{6}A modification peaks (5’-3’)
CD40_peak wild-type	TCTGAGGACTGCTTG
CD40_peak mutation	TCTGAGGGCTGCTTG
CD80_peak wild-type	GAACCGGACTTTATA
CD80_peak mutation	GAACCGTACTTTATA
Tirap_peak wild-type	TCAAGGGACTGTGCT
Tirap_peak mutation	TCAAGGGGCTGTGCT

Supplementary Table 5 Oligo sequences synthesized targeting Ythdf1

Gene	5'	Stem	Loop	Stem	3'
Ythdf1	Ccgg	cgACAACAAACCTGTCACA	CTCGAG	TTGTGACAGGTTTGTTGTCG	TTTTTg
	aattcaaaaa	cgACAACAAACCTGTCACAAA	CTCGAG	TTGTGACAGGTTTGTTGTCG	

Supplementary Table 6 qPCR primers used for detecting expression of indicated molecules

Primers	Sequence (5’-3’)
β-actin_F	AGTGTGACGTTGACATCCGT
β-actin_R	GCAGCTCAGTAACAGTCCGC
Mettl3_F	CTGGGCACTTGGATTTAAGGAA
Mettl3_R	TGAGAGGTGGTGTAGCAACTT
IL-6_F	TAGTCCTTCCTACCCCAATTTCC
IL-6_R	TTGGTCCTTAGCCACTCCTTC
IL-12_F	CTCCTAAACCACCTCAGTTTG
IL-12_R	CAGGAATAATGTTTCAGTTTTTC
H2-Eb2_F	TGCTCTGTGATTGACTTCTAC
H2-Eb2_R	GGCAGGTGTAAACCTCTCC
Luci_F	TCTGTGATTTGTATTCAGCCCAT
Luci_R	GCTATGAAGAGATACGCCCTG
Ren_F	GCGAAGAGGGCGAGAAGATG
Ren_R	TTCTTTGAAGGGTTCCAGGTAG
CD80_Flag_F	GATTACAAGGATGACGACG
CD80_Flag_R	AGGCACAGTCGAGGCTGA
GAPDH_F	CTGGGCTACACTGAGCACC
GAPDH_R	AAGTGGTCGTTGAGGGCAATG
Itgam_F	ATGGACGCTGATGGCAATACC
Itgam_R	TCCCCATTCACGTCTCCCA
Ctla4_F	GAGTCTGTGTGGGTTCAAAC
Ctla4_R	AAAAGAAGAGTGAGCAGGGC
*CD80(531)_F	CATTGCTGGGAAACTAAAAG
*CD80(531)_R	CAGGATGATAAGAGAGTAGG
*CD40(1002)_F	TGGCTCTGAGGACTGCTTG
*CD40(1002)_R	TGATAGGGGGCAGGCATGA
*Tirap(1508)_F	ACTCCCCCAGGAAGACAACG
*Tirap(1508)_R	GATGCCAGAGGAAGAAGACAG
*Socs3(1547)_F	AGATTTCGCTTCGGGACTAG
*Socs3(1547)_R	GGAGCCAGCGTGGATCTGC

*, number in the bracket represent the location of the m^6A modified A in the mRNA.
