# Supplementary material for: Mettl3-mediated mRNA m6A methylation promotes dendritic cell activation
Source: Nat Commun. 2019 Apr 23;10:1898. doi: 10.1038/s41467-019-09903-6 (PMC6478715; doi:10.1038/s41467-019-09903-6)
Supplement: Supplementary file 3 — Reporting Summary [file 41467_2019_9903_MOESM3_ESM.pdf]

nature research

Corresponding author(s): Xuetao Cao

Last updated by author(s): 2019-03-15

Reporting Summary

Nature Research wishes to improve the reproducibility of the work that we publish. This form provides structure for consistency and transparency in reporting. For further information on Nature Research policies, see Authors & Referees and the Editorial Policy Checklist.

Please do not complete any field with "not applicable" or n/a. Refer to the help text for what text to use if an item is not relevant to your study.
For final submission: please carefully check your responses for accuracy; you will not be able to make changes later.

Statistics

For all statistical analyses, confirm that the following items are present in the figure legend, table legend, main text, or Methods section.

n/a	Confirmed	
☐	☒	The exact sample size (*n*) for each experimental group/condition, given as a discrete number and unit of measurement
☐	☒	A statement on whether measurements were taken from distinct samples or whether the same sample was measured repeatedly
☐	☒	The statistical test(s) used AND whether they are one- or two-sided<br>*Only common tests should be described solely by name; describe more complex techniques in the Methods section.*
☒	☐	A description of all covariates tested
☐	☒	A description of any assumptions or corrections, such as tests of normality and adjustment for multiple comparisons
☐	☒	A full description of the statistical parameters including central tendency (e.g. means) or other basic estimates (e.g. regression coefficient) AND variation (e.g. standard deviation) or associated estimates of uncertainty (e.g. confidence intervals)
☐	☒	For null hypothesis testing, the test statistic (e.g. *F*, *t*, *r*) with confidence intervals, effect sizes, degrees of freedom and *P* value noted<br>*Give P values as exact values whenever suitable.*
☒	☐	For Bayesian analysis, information on the choice of priors and Markov chain Monte Carlo settings
☒	☐	For hierarchical and complex designs, identification of the appropriate level for tests and full reporting of outcomes
☒	☐	Estimates of effect sizes (e.g. Cohen's *d*, Pearson's *r*), indicating how they were calculated

Our web collection on statistics for biologists contains articles on many of the points above.

Software and code

Policy information about availability of computer code

Data collection	For meRIP-seq test, data were detected using Tape Station 2200 system; For mRNA mass spectra test, data were collected using G3335AA MassHunter Qualitative Analysis Software(Agilent 1290 UPLC);For ribosome separating test, the expected ribosome fraction was acquired using flocell software (Piston Gradient Fractionator system);For dual luciferse report,data were recorded by Gene5 sotware(Promega reporter assay system).
Data analysis	Classic Excel was used for T-test; For FACS, BD FACSDiva and Flowjo software were used; For western blotting quantification of band was carried out by ImageJ software; Software and packages analyzing meRIP sequencing data were described under"m6A-meRIP-Seq and m6A-meRIP qPCR" section in supplementary methods.

For manuscripts utilizing custom algorithms or software that are central to the research but not yet described in published literature, software must be made available to editors/reviewers. We strongly encourage code deposition in a community repository (e.g. GitHub). See the Nature Research guidelines for submitting code & software for further information.

Data

Policy information about availability of data

All manuscripts must include a data availability statement. This statement should provide the following information, where applicable:
- Accession codes, unique identifiers, or web links for publicly available datasets
- A list of figures that have associated raw data
- A description of any restrictions on data availability

High-throughput sequencing data can be accessed in the Gene Expression Omnibus under accession number GSE108333.

Field-specific reporting

Please select the one below that is the best fit for your research. If you are not sure, read the appropriate sections before making your selection.

☒ Life sciences ☐ Behavioural & social sciences ☐ Ecological, evolutionary & environmental sciences

Life sciences study design

All studies must disclose on these points even when the disclosure is negative.

Sample size	Required sample sizes were decided based on experience and preliminary experiments.
Data exclusions	No data were excluded.
Replication	All high-throughput sequencing experiments were performed twice, and other assays were performed with no less than three replicates.
Randomization	All animal- and cell-based samples in each of the group were included and no method of randomization was applied.
Blinding	Binding is not relevant to our study, as we need to know the genotypes of the mouse strains.

Reporting for specific materials, systems and methods

We require information from authors about some types of materials, experimental systems and methods used in many studies. Here, indicate whether each material, system or method listed is relevant to your study. If you are not sure if a list item applies to your research, read the appropriate section before selecting a response.

Materials & experimental systems

n/a	Involved in the study	
☐	☒	Antibodies
☐	☒	Eukaryotic cell lines
☒	☐	Palaeontology
☐	☒	Animals and other organisms
☒	☐	Human research participants
☒	☐	Clinical data

Methods

n/a	Involved in the study	
☒	☐	ChIP-seq
☐	☒	Flow cytometry
☒	☐	MRI-based neuroimaging

Antibodies

Antibodies used	Antibodies for meRIP experiments: Rabit anti-m6A polyclonalantibdy(Synaptic Systems,Cat.No.202003); Antibodies for western blotting: anti-Mettl3(Proteintech,15073-1-AP,1:1000),anti-Mettl14(Sigma,HPA0380021:1000),anti-Fto(Abcam,ab124892,1:1000),anti-Wtap(Abcam,ab118339,1:500), anti-p65 phosphorylated at Ser536(CST,3031S,1:1000),anti-IKKα-IKKβ phosphorylated at Ser176 and Ser180(CST,2697S,1:1000),antibody to Erk phosphorylated at Thr202 and Tyr204(9106S,1:1000),anti-Jnk phosphorylated at Thr183 and Tyr185(CST,9255S,1:1000),anti-β-actin (CST,3700S,1:10000),anti-Flag-HRP (CST,2044S,1:2000),anti-p65 (CST,6956S,1:1000),anti-p38 (CST,9212S,1:1000),anti-IκBα (CST,9242S,1:1000),anti-IKKβ(CST,8943S,1:1000);Antibodies for FACS: PE Hamster anti-mouse CD11c(BD Biosciences,553802), PerCP-Cy5.5 Hamster anti-mouse CD80(BD Biosciences,560526), PerCP-Cy5.5 anti-mouse CD86(Biolegend,105027),PE-Cy7 anti-mouse CD40(Biolegend,124621), PE-Cy7 anti-mouse I-Ab(Biolegend,116420),PE-Cy7 anti-mouse CD4 (Biolegend,100422),APC anti-mouse TCR Vβ5.1 antibody(Biolegend,139511), FITC anti-mouse CD45.2 Antibody(Biolegend,109805).
Validation	All the primary antibodies were validated by the manufacturers. Antibodies for meRIP experiments were validated by previous study (Nature, 563, 249-253, doi.org/10.1038/s41586-018-0666-1).

Eukaryotic cell lines

Policy information about cell lines

Cell line source(s)	The HEK293T cells used in dual luciferase report assay and RIP-seq were purchased from ATCC.
Authentication	None of the cell lines have been authenticated.
Mycoplasma contamination	The cell lines were tested negative for mycoplasma contamination.
Commonly misidentified lines (See ICLAC register)	The cell lines used are not listed in the database of ICLAC.

Animals and other organisms

Policy information about studies involving animals; ARRIVE guidelines recommended for reporting animal esearch

Laboratory animals	The wild-type C57BL/6,OT-II mice (which have transgenic expression of a T cell antigen receptor specific for chicken ovalbumin amino acids 323–339 (OVA(323-339)),CD45.1^{+} congenic mice and Mettl3$^{fl/fl}$CD11c-Cre mice were used. For in vivo immuniztion experiment, the OT-II mice,CD45.1^{+} congenic mice and Mettl3$^{fl/fl}$CD11c-Cre mice were used; For meRIP-seq, the wild-type C57BL/6;For RNA lifetime and ribosome profile assays, the Mettl3$^{fl/fl}$CD11c-Cre mice were used.
Wild animals	No wild animals were used.
Field-collected samples	No Field-collected samples were used.
Ethics oversight	All animal experiments were carried out according to National Institute of Health Guide for the Care and Use of Laboratory Animals, with the approval of the Scientific Investigation Board of Second Military Medical University.

Note that full information on the approval of the study protocol must also be provided in the manuscript.

Flow Cytometry

Plots

Confirm that:

- [ ] The axis labels state the marker and fluorochrome used (e.g. CD4-FITC).
- [x] The axis scales are clearly visible. Include numbers along axes only for bottom left plot of group (a 'group'is an analysis of identical Markers).
- [x] All plots are contour plots with outliers or pseudocolor plots.
- [x] A numerical value for number of cells or percentage (with statistics) is provided.

Methodology

Sample preparation	For splenic DC, the whole spleen were grinded and filtered by 40μM nylon mesh filter to prepare single cell suspensions and the red blood cells were removing using Tris-NH4Cl, the CD11c^{+} DCs were separated after incubating with anti-CD11c microbeads for 15min; For BMDC, BM separated from mice were cultured in RPMI-1640 medium and differentiated to DC by adding GM-CSF and IL-4 ("BMDC preparation" in Supplementary methods); For splenic T cells , the whole spleen were grinded to single cells and the red blood cells were removing using Tris-NH4Cl, the CD4+ T cells were separated after incubating with anti-CD4 microbeads for 15min.
Instrument	BD LSR II and LS Fortessa
Software	BD FACSDiva and Flowjo software was used to analyze the data.
Cell population abundance	An aliquot of the sorted CD11c DC or CD4 T cells were always collected and run on a cytometer to verify purity of the samples collected. In addition, cell counts were performed on samples post sort to verify correct cell numbers.
Gating strategy	Dendritic cells were identified from whole spleen first by discrimination of cells by size (FSC-A by SSC-A). Following this, CD11c, I-Ab double positive cells were selected for phenotypic and percentage analysis; For analyzing the capacity of DC priming T cells activation in vivo, cells from whole lymph nodes were first gated by discrimination of cells by size (FSC-A by SSC-A) and followed by CD4 positive gate, then CD45.2 positive cells were further gated. For lentivirus transfection assay, dendritic cells were first discriminated by size (FSC-A by SSC-A). Then cells positive for both GFP and CD11c were gated for phenotypic analysis or for cell sorting .

- [x] Tick this box to confirm that a figure exemplifying the gating strategy is provided in the Supplementary Information.

This checklist template is licensed under a Creative Commons Attribution 4.0 International License, which permits use, sharing, adaptation, distribution and reproduction in any medium or format, as long as you give appropriate credit to the original author(s) and the source, provide a link to the Creative Commons license, and indicate if changes were made. The images or other third party material in this article are included in the article's Creative Commons license, unless indicated otherwise in a credit line to the material. If material is not included in the article's Creative Commons license and your intended use is not permitted by statutory regulation or exceeds the permitted use, you will need to obtain permission directly from the copyright holder. To view a copy of this license, visit http://creativecommons.org/licenses/by/4.0/
