# Supplementary material for: Mettl3-mediated mRNA m6A methylation promotes dendritic cell activation
Source: Nat Commun. 2019 Apr 23;10:1898. doi: 10.1038/s41467-019-09903-6 (PMC6478715; doi:10.1038/s41467-019-09903-6)
Supplement: Supplementary file 2 — Peer Review File [file 41467_2019_9903_MOESM2_ESM.pdf]

Editorial Note: This manuscript has been previously reviewed at another journal that is not operating a transparent peer review scheme. The manuscript was considered suitable for publication without further review at *Nature Communications.*
